# All-Solid-State Lithium–Sulfur Batteries: Recent Progress, Challenges, and Perspectives

**DOI:** 10.3390/ma19122565

**Published:** 2026-06-13

**Authors:** Yoonha Hwang, Yeo Jin An, Soohyun Sim, Changhoon Choi, Minjeong Shin

**Affiliations:** 1 Department of Chemistry, Sungshin Women’s University, 55, Dobong-ro 76 ga-gil, Gangbuk-gu, Seoul 01133, Republic of Korea; prettycat4637@gmail.com (Y.H.);; 2 School of Chemistry and Energy, Sungshin Women’s University, 55, Dobong-ro 76 ga-gil, Gangbuk-gu, Seoul 01133, Republic of Korea; 3 Department of Materials Science and Engineering, Sungshin Women’s University, 55, Dobong-ro 76 ga-gil, Gangbuk-gu, Seoul 01133, Republic of Korea; 4 Center for NanoBio Applied Technology, Sungshin Women’s University, 55, Dobong-ro 76 ga-gil, Gangbuk-gu, Seoul 01133, Republic of Korea

**Keywords:** all-solid-state lithium–sulfur batteries, all-solid-state batteries, lithium–sulfur batteries, solid electrolytes, composite cathodes, interface engineering, interface stabilization, polysulfide suppression, solid electrolyte interphase

## Abstract

All-solid-state lithium–sulfur batteries (ASSLSBs) couple the high theoretical energy density of sulfur (2600 Wh kg^−1^) with the safety and polysulfide-shuttle suppression advantages of solid electrolytes (SEs). In practice, however, sluggish solid-state conversion kinetics, chemo-mechanical degradation in composite cathodes, and large solid–solid interfacial resistance remain the principal barriers to practical implementation. This review systematically examines recent progress across the three key components of ASSLSBs: cathodes, solid electrolytes, and interfaces. For cathodes, S/C composite design strategies and alternative active materials—including Li_2_S, metal sulfides, and organosulfur compounds—are discussed. For solid electrolytes, inorganic (sulfide, oxide, halide, and hydride), polymer, and hybrid composite systems are compared. For interfaces, physical strategies (stack pressure, compliant interlayers, three-dimensional cathode architectures) and chemical strategies (cathode–SE and Li metal–SE interphase engineering, in situ stabilization) are evaluated. Outstanding challenges and design guidelines for next-generation ASSLSBs are discussed.

## 1. Introduction

### 1.1. Limitations of Conventional Lithium–Sulfur Batteries

The rapid growth of electric vehicles (EVs) and grid-scale energy storage has intensified the need for batteries that exceed the energy density of current lithium-ion batteries (LIBs) [1,2]. State-of-the-art LIBs based on intercalation cathodes such as LiNi_x_Mn_y_Co_(1−x−y)_O_2_ (NMC) deliver practical gravimetric energy densities of 250–300 Wh kg^−1^, which are approaching the limits set by their intercalation chemistry [3,4]. Lithium–sulfur (Li–S) batteries offer a theoretical specific capacity of 1675 mAh g^−1^ and a theoretical energy density of 2600 Wh kg^−1^, making them among the most attractive candidates for next-generation energy storage [5,6,7]. Sulfur is also earth-abundant and inexpensive, adding practical appeal alongside the performance advantages [8,9].

In practice, however, Li–S batteries with liquid electrolytes suffer from several interrelated problems that have prevented commercialization. The most well-known is the polysulfide shuttle effect. During discharge, elemental sulfur is reduced to higher-order lithium polysulfides (Li_2_S_x_, 4 ≤ x ≤ 8), which dissolve readily in ether-based electrolytes and diffuse to the lithium metal anode [10,11]. There, they are chemically reduced to shorter-chain polysulfides, which migrate back to the cathode and are reoxidized—a parasitic cycle that causes irreversible active material loss, rapid capacity fading, and low Coulombic efficiency [12,13,14].

Extensive electrolyte engineering efforts—including functional additives such as LiNO_3_ [15] high-concentration electrolytes (HCEs) [16], and localized HCEs (LHCEs) [17,18,19]—have been developed to mitigate the shuttle effect, though polysulfide dissolution cannot be eliminated entirely because the thermodynamic driving force for solvation persists regardless of electrolyte formulation.

Both elemental sulfur (~5 × 10^−30^ S cm^−1^) and the final discharge product Li_2_S (~10^−13^ S cm^−1^) are electronic insulators, requiring intimate contact with a conductive carbon host to sustain electrochemical activity across the cathode [8,20]. Sulfur also undergoes ~80 vol% expansion upon full lithiation, which can crack the electrode structure and disconnect active material from the conductive network [21,22]. At the anode, lithium metal is prone to dendritic growth during cycling, which poses short-circuit and safety risks [23,24]. The volatility and flammability of conventional ether-based electrolytes exacerbate thermal runaway hazards, a concern that becomes critical in large-format cell designs [25,26].

### 1.2. All-Solid-State Lithium–Sulfur Batteries: Concept and Advantages

**Figure 1:**
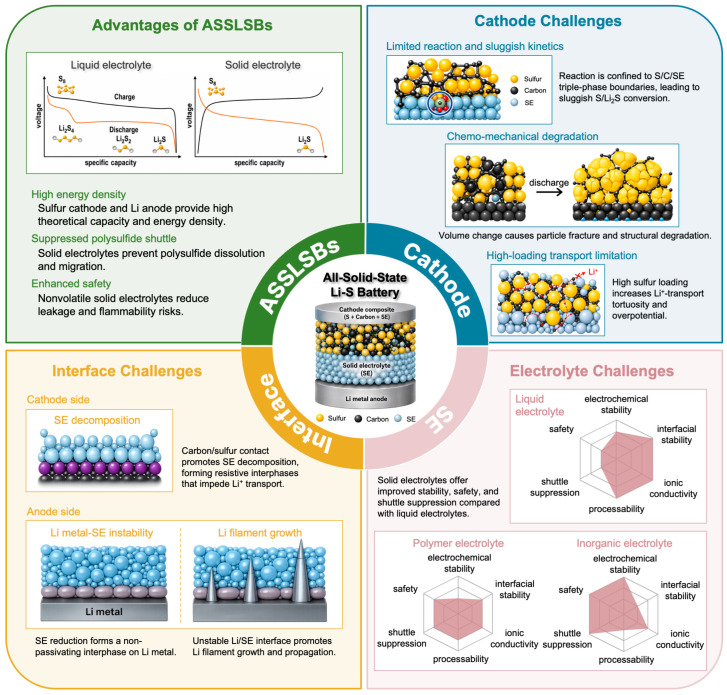
Schematic overview of ASSLSB advantages and key challenges. ASSLSBs offer high energy density, suppressed polysulfide shuttle, and improved safety by replacing liquid electrolytes with solid electrolytes. Key challenges arise from sluggish cathode reaction kinetics, chemo-mechanical degradation, solid electrolyte trade-offs, and unstable electrode–electrolyte interfaces, including SE decomposition and Li filament growth.

Replacing the liquid electrolyte with a solid electrolyte SE addresses several of the limitations described above (Figure 1) [9,21,27]. ASSLSBs retain the high-capacity sulfur cathode and lithium metal anode while eliminating the liquid phase entirely. Because SEs are non-volatile and thermally stable well above the decomposition temperature of organic solvents, ASSLSBs can be safer and less susceptible to thermal runaway compared with their liquid counterpart, particularly when fully solid, nonflammable electrolytes are used [26,28].

The primary advantage of ASSLSBs is the suppression of polysulfide dissolution. Without a solvent medium, polysulfides cannot dissolve and migrate between electrodes. In cells based on dense inorganic SEs, the sulfur redox chemistry is generally believed to proceed via a solid–solid reaction pathway: S_8_ is thought to be converted directly to Li_2_S_2_ and Li_2_S without generating soluble polysulfide intermediates [28,29]. This eliminates the shuttle effect, confines active material within the cathode, and enables higher Coulombic efficiencies [9]. The absence of a liquid electrolyte also removes the need for a separator, simplifying cell architecture and improving the ratio of active to inactive material in the cell stack [30].

The scientific foundation for ASSLSBs was established in 2003, when Hayashi et al. first demonstrated an all-solid-state Li/S cell using a sulfur–CuS composite cathode and a Li_2_S–P_2_S_5_ glass-ceramic SE, achieving reversible capacities exceeding 650 mAh g^−1^ over 20 cycles at room temperature [31]. Only limited progress was achieved in the 2000s, largely constrained by the low ionic conductivities of available SEs. The field was substantially accelerated by the discovery of superionic sulfide conductors, most notably Li_10_GeP_2_S_12_ (LGPS) in 2011 with a room-temperature conductivity of 12 mS cm^−1^ [32], followed by the development of argyrodite-type Li_6_PS_5_Cl (LPSCl) and halide SEs, which collectively expanded the palette of viable SE materials and enabled cathode composites with improved ionic transport. Over the past decade, research output in the ASSLSB field has grown exponentially, with investigations now spanning cathode composite design, SE development across sulfide, oxide, and halide families, interfacial engineering, and cell-level integration, as reflected by the emergence of comprehensive reviews from multiple groups [9,21,26,28,29,33,34,35].

Despite these advantages, the transition to a fully solid-state architecture introduces a new set of materials and engineering challenges that are distinct from those encountered in liquid electrolyte Li–S cells and that must be addressed before the theoretical benefits of ASSLSBs can be realized in practice.

### 1.3. Key Challenges in All-Solid-State Lithium–Sulfur Batteries

While the solid-state configuration resolves polysulfide dissolution, it introduces a distinct set of challenges that are specific to the ASSLSB architecture and that go beyond those common to solid-state batteries in general (Figure 1).

#### 1.3.1. Cathode

Because both sulfur and the discharge product Li_2_S are electronic and ionic insulators, the cathode must be formulated as a composite in which carbon provides electronic conductivity and SE particles provide ionic pathways, with the electrochemical reaction confined to the triple-phase boundary where all three components meet [29,33]. As a result, the density, continuity, and stability of these reaction boundaries directly determine sulfur utilization, polarization, and rate capability.

The large volumetric expansion (~80 vol%) associated with the S_8_/Li_2_S conversion imposes severe mechanical stress on the rigid solid matrix. As this expansion reverses on delithiation, the cathode is repeatedly cycled between swollen and contracted states, and the accumulated strain induces irreversible contact loss among sulfur, carbon, and SE particles, resulting in chemo-mechanical degradation. The strain also opens voids and cracks at the particle interfaces, which steadily degrade the percolated Li^+^ and electron pathways and increase cell impedance. Unlike liquid-electrolyte cells, these fractured or disconnected interfaces cannot be re-wetted by a liquid phase, making contact preservation a central design requirement in ASSLSB cathodes [36,37].

In the absence of soluble polysulfide intermediates, ASSLSBs convert S_8_ to Li_2_S directly at solid–solid contacts, which eliminates the shuttle effect but also removes the solution-mediated mass transport that accelerates sulfur redox in liquid cells [9,33]. This solid-state reaction is intrinsically slow. Li_2_S is both an electronic and ionic insulator, and its low bulk Li^+^ diffusion coefficient makes ion transport through the growing product layer rate-limiting [38]. Charge transfer at the solid–solid interface further demands a high activation energy, because Li_2_S must nucleate and grow directly from S_8_ without the soluble intermediates that lower the barrier in liquid cells. As Li_2_S accumulates, it passivates the reaction sites and progressively shrinks the accessible reaction area, slowing the conversion over the course of discharge.

Increasing sulfur content to improve energy density simultaneously lengthens and increases the tortuosity of the ionic transport pathways within the composite, raising overpotential and limiting rate capability—a trade-off between transport and energy density that has no direct analog in liquid electrolyte systems [21,33]. The choice of active material further shapes the design space: elemental sulfur (S_8_) offers the highest theoretical capacity but requires a lithium metal anode, while pre-lithiated Li_2_S is compatible with lithium-free anode configurations and avoids the initial volume expansion upon lithiation, though it introduces additional challenges related to air sensitivity and high initial interfacial resistance [25,29].

#### 1.3.2. Solid Electrolytes

Each class of SE involves trade-offs between ionic conductivity, electrochemical stability, and processability. Sulfide-based SEs—including Li_3_PS_4_ [39], LGPS [32], and LPSCl (argyrodite) [40]—are the most widely used in ASSLSBs owing to their high room-temperature ionic conductivities (~10^−2^ S cm^−1^) and compatibility with cold pressing, but their electrochemical stability windows are narrow and they are reactive with both lithium metal and carbon additives. Oxide-based SEs such as Li_7_La_3_Zr_2_O_12_ (LLZO) [41] offer superior chemical stability but require high-temperature sintering and exhibit poor interfacial contact with composite cathodes. Polymer SEs such as poly(ethylene oxide) (PEO) are mechanically compliant and conformally contact electrodes, but their room-temperature conductivity typically falls below 10^−5^ S cm^−1^, restricting rate performance [42,43]. Halide SEs and hybrid composite electrolytes have emerged as promising alternatives that seek to balance conductivity, stability, and processability, though their integration with sulfur cathodes remains under active development [44,45].

#### 1.3.3. Electrode–Electrolyte Interfaces

The replacement of a liquid electrolyte with a rigid solid fundamentally changes the nature of electrode–electrolyte contact. Unlike liquids, which conformally wet porous electrode structures, solid electrolytes make only point and area contacts with electrode particles, generating interfacial voids that impede ionic charge transfer [9,46]. Beyond the physical contact problem, the high SE surface area within the composite cathode promotes electrochemical SE decomposition, forming ionically resistive interphases at the SE–carbon and SE–sulfur interfaces that deteriorate ion transport upon cycling [33,47,48]. This creates a dilemma intrinsic to ASSLSB cathode design: finer composite mixing improves the triple-phase contact and active material utilization but simultaneously enlarges the reactive SE surface area and accelerates interfacial degradation [33].

At the lithium metal anode, sulfide-based SEs are thermodynamically unstable against metallic lithium and undergo reductive decomposition to form mixed ionic–electronic interphase products such as Li_2_S and Li_3_P, which—unlike a passivating solid electrolyte interphase (SEI) in liquid electrolyte cells—allow continuous SE decomposition and irreversible Li consumption upon cycling [49,50]. Lithium stripping generates interfacial voids that concentrate local current density and promote non-uniform plating, while lithium filaments have been observed to propagate through grain boundaries and defects of ceramic SEs under practical cycling conditions—contrary to early assumptions that dense inorganic SEs would mechanically suppress dendrite growth [24,51].

#### 1.3.4. Processing and Scalability

Most inorganic SEs are moisture-sensitive and require dry-room or inert-atmosphere handling throughout fabrication [27]. Cell assembly typically relies on high-pressure uniaxial or isostatic pressing to establish adequate solid–solid contact, which is incompatible with the roll-to-roll manufacturing processes used in conventional battery production [21,26]. Bridging the gap between laboratory-scale pellet cells and industrially relevant pouch or prismatic formats remains a critical challenge for the field.

### 1.4. Scope and Organization of This Review

This review is organized by component—cathode, solid electrolyte, and electrode–electrolyte interface—with each section structured to reflect the principal design strategies and material classes currently under investigation.

Section 2 covers cathode design. Section 2.1 examines S/C composite cathodes, from conventional ball-milled composites (Section 2.1.1) to porous and geometrically structured carbon hosts (Section 2.1.2) and surface-engineered carbon networks (Section 2.1.3). Section 2.2 surveys alternative cathode chemistries, including Li_2_S-based cathodes (Section 2.2.1), metal sulfide cathodes (Section 2.2.2), and organosulfur materials and molecular redox mediators (Section 2.2.3).

Section 3 reviews solid electrolytes by material class. Section 3.1 covers inorganic SEs—sulfide (Section 3.1.1), oxide (Section 3.1.2), halide (Section 3.1.3), and hydride (Section 3.1.4). Section 3.2 discusses solid polymer electrolytes (Section 3.2.1) and gel polymer electrolytes (Section 3.2.2). Section 3.3 examines hybrid composite electrolytes.

Section 4 addresses interface engineering. Section 4.1 covers physical strategies, including stack pressure and current-collector surface engineering, compliant interlayers, and three-dimensional cathode architectures. Section 4.2 discusses chemical interface engineering at cathode–SE and Li metal–SE interfaces, as well as in situ interphase formation strategies.

Section 5 summarizes key findings and identifies remaining challenges and design priorities for advancing ASSLSBs toward practical application. Table 1 compiles representative cell configurations from across these sections, providing a comparative overview of the reported cathode composites, solid electrolytes, and cell-level performance.

## 2. Cathodes

The cathode constraints introduced in Section 1.3—poor electronic and ionic conductivity of both S and Li_2_S, the ∼80 vol% expansion during full lithiation, the slow kinetics of solid-state S/Li_2_S conversion, and the trade-off between high sulfur loading and continuous ionic percolation—have motivated two broad cathode design strategies for ASSLSBs. The first relies on elemental sulfur dispersed within conductive carbon hosts, in which the carbon framework provides electronic pathways and SE particles supply Li^+^-transport channels. Sustaining percolated reaction boundaries through controlled architecture, porosity, and surface chemistry is therefore the central objective of S/C composite cathode design (Section 2.1). The second strategy employs alternative composite cathodes, including Li_2_S-based cathodes, metal sulfides, and other functional sulfur-containing systems, to address the kinetic, mechanical, and interfacial limitations of conventional S/C cathodes (Section 2.2).

### 2.1. Sulfur/Carbon (S/C) Composite Cathodes

#### 2.1.1. Conventional S/C Composites and Solid-State Reaction Boundaries

In liquid-electrolyte Li–S batteries, porous carbon hosts primarily serve to confine sulfur, provide electronic conductivity, and mitigate polysulfide dissolution, while ionic transport is more readily maintained by liquid electrolyte infiltration into the porous electrode. In ASSLSBs, this passive role of carbon is insufficient. Because no liquid phase is available to wet the cathode composite, Li^+^ transport must be built directly into the electrode through intimate mixing of sulfur, conductive carbon, and SE particles. Electrochemical activity is confined to triple-phase boundaries, whose density and continuity govern sulfur utilization, polarization, and rate capability.

In addition, inorganic SEs impose a solid–solid sulfur conversion pathway. Unlike liquid cells, where soluble polysulfide intermediates assist mass transport, ASSLSBs rely predominantly on direct S_8_/Li_2_S conversion at solid–solid interfaces. This configuration eliminates the shuttle effect but also removes the solution-mediated transport pathway that facilitates sulfur redox kinetics in liquid electrolytes. Therefore, the fundamental design requirement for ASSLSB cathodes is to achieve intimate and mechanically stable contact among sulfur, carbon, and SE throughout the electrode volume.

**Figure 2:**
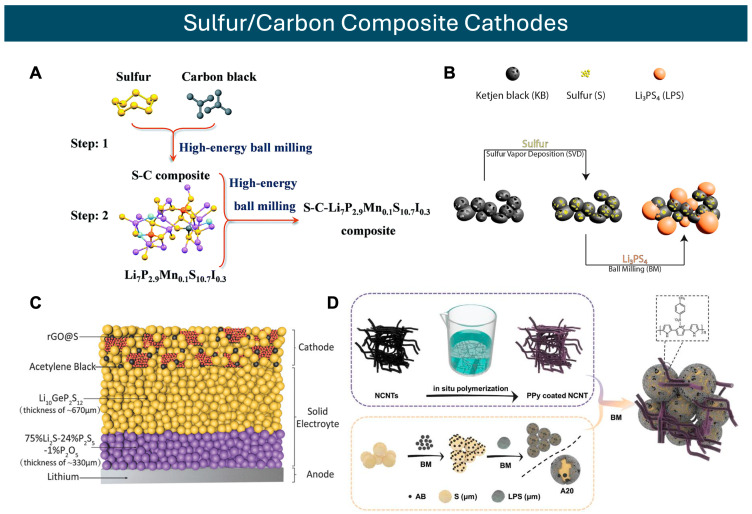
Design strategies for sulfur/carbon composite cathodes in ASSLSBs. (**A**) Schematic illustration of conventional S/C/SE composite cathodes prepared by sequential high-energy ball milling (Reproduced with permission from Ref. [52], copyright 2017 Royal Society of Chemistry). (**B**) Sulfur vapor deposition into Ketjen black followed by ball milling with Li_3_PS_4_ for improved sulfur confinement and solid–solid contact (Reproduced with permission from Ref. [59], copyright 2021 American Chemical Society). (**C**) rGO-supported nanosulfur cathode incorporated into an LGPS-based ASSLSB configuration (Reproduced with permission from Ref. [60], copyright 2017 Wiley-VCH). (**D**) Polypyrrole-coated N-doped carbon nanotube networks for enhancing electronic connectivity and mechanical compliance in S/C/SE cathodes (Reproduced with permission from Ref. [61], copyright 2024 Wiley-VCH).

Early studies established mechanical mixing as the baseline approach for constructing such composite cathodes (Figure 2A) [52]. Nagao et al. prepared elemental sulfur, acetylene black, and Li_2_S–P_2_S_5_ glass-ceramic SE composites by planetary ball milling, which reduced sulfur particle size, increased interfacial contact, and enabled reversible room-temperature cycling with high Coulombic efficiency [53]. Subsequent work demonstrated that performing the milling step above sulfur’s melting point allowed molten S to coat carbon surfaces prior to solidification, which improved S–C contact and ultimately raised sulfur utilization relative to room-temperature milling [54]. The importance of cathode composition was further clarified by Nagata and Chikusa, who demonstrated that the S/C/SE ratio strongly affects the balance among active-material loading, electronic conduction, ionic transport, and rate capability [55,56]. Related early reports on mechanically mixed S/C/SE cathodes similarly emphasized the importance of cathode composition, particle size, and triple-phase contact density in determining sulfur utilization and rate capability [31,57,58].

Taken together, these studies established that the performance of conventional S/C composite cathodes is governed by the accessibility and continuity of solid-state reaction boundaries, which are determined by the mixing method, particle size, and component ratio. However, simple mechanical mixing provides limited control over sulfur distribution and interfacial continuity, motivating the development of structured carbon hosts and more advanced cathode architectures. The central design principle at this baseline level is to maximize triple-phase contact while maintaining sufficient electronic and ionic percolation—a balance that mechanical mixing can establish but only coarsely control.

#### 2.1.2. Porous and Structured Carbon Hosts for Sulfur Confinement and Volume Accommodation

The limitations of ball-milled S/C/SE composites, including limited control over sulfur distribution, insufficient solid–solid contact, and mechanical degradation caused by unconstrained volume changes, motivated the use of carbon hosts with engineered porosity and architecture. In these designs, the carbon structure provides electronic conductivity, confines sulfur within defined spaces, and accommodates the volume change during S/Li_2_S conversion without severe loss of contact with the SE.

Porous carbon hosts were among the first structural solutions explored for ASSLSB cathodes. Nagao et al. demonstrated that incorporating mesoporous carbon into S/SE composites improved sulfur utilization and cycling stability relative to carbon black-based electrodes by increasing the interfacial contact area among sulfur, carbon, and the SE [62]. Sakuda et al. extended this approach by using carbon with interconnected mesopores of approximately 5 nm, showing that a continuous mesoporous network increased ion-accessible reaction sites and improved rate capability; the resulting cells delivered 1100 mAh g^−1^ after 400 cycles at 1.3 mA cm^−2^ at 25 °C [63]. Alzahrani et al. further showed that the sulfur infiltration method critically determines confinement quality [59]. Sulfur vapor deposition (SVD) into Ketjen Black produced smaller and more uniformly distributed sulfur domains than liquid or solid deposition routes, while providing sufficient pore volume to accommodate both sulfur and fully lithiated Li_2_S (Figure 2B) [59]. The SVD-derived composite delivered 1792.6 mAh g^−1^ at 0.1 C and maintained 957.3 mAh g^−1^ at a sulfur loading of 4.5 mg cm^−2^, although the exceptionally high capacity was partly associated with additional contribution from the sulfide SE [59].

One-dimensional carbon frameworks offer a complementary approach in which interconnected conductive networks, rather than discrete pores, sustain electronic transport throughout the composite cathode. Zhang et al. deposited sulfur onto carbon nanotube (CNT) surfaces and dispersed the CNT@S composite in an LGPS electrolyte matrix, achieving 1193 mAh g^−1^ at 0.1 C and 396 mAh g^−1^ at 5 C at 60 °C [64]. The improved rate capability was attributed to the continuous one-dimensional electronic network maintained by the CNT framework. Phuc et al. used carbon nanofibers (CNF) as a structural backbone and showed that the one-dimensional CNF geometry establishes extended line contacts with SE particles, rather than isolated point contacts, thereby reducing interfacial resistance and enabling full sulfur utilization at 0.1 C and approximately 600 mAh g^−1^ at 1 C [65].

Two-dimensional carbon structures manage volume changes through uniform spatial distribution of sulfur and planar deformation rather than pore confinement alone. In the work of Yao et al., an ∼2 nm conformal amorphous-sulfur shell was deposited onto reduced graphene oxide (rGO) sheets, and the resulting rGO@S nanocomposite was then dispersed throughout an LGPS/acetylene-black matrix to provide simultaneous Li^+^ and e^−^ percolation (Figure 2C) [60]. The thin sulfur coating shortened Li^+^ diffusion distances, maximized contact area with the SE, and distributed the volume change more uniformly across the conductive rGO framework. As a result, the cell maintained 830 mAh g^−1^ at 1 C for 750 cycles at 60 °C [60].

The above examples indicate that carbon-host geometry and sulfur-loading methodology strongly influence sulfur confinement, electronic percolation, volume-change tolerance, and the accessibility of solid-state reaction pathways in ASSLSB cathodes. In addition to the representative examples discussed above, mesoporous carbons, carbon nanotubes, carbon nanofibers, graphene derivatives, and hierarchical carbon frameworks have been broadly examined to balance sulfur confinement, electronic connectivity, and solid-state Li^+^ accessibility [64,66,67,68,69]. As a design principle, no single geometry is universally optimal: porous hosts favor sulfur confinement, one-dimensional networks favor electronic percolation, and two-dimensional architectures favor uniform volume accommodation, so the carbon host should be matched to the dominant limitation of the target cell, or combined in hierarchical frameworks.

#### 2.1.3. Surface-Engineered Carbon Networks for Solid–Solid Contact

Whereas Section 2.1.2 addressed carbon-host geometry as a means of controlling sulfur confinement and volume accommodation, the surface chemistry and network functionality of carbon remain separate design considerations. The intrinsically nonpolar character of carbon can weaken its affinity toward sulfur/Li_2_S and sulfide SE particles, promoting active-material detachment during cycling, while its high electronic conductivity can accelerate oxidative decomposition of adjacent SEs. Surface and network engineering of carbon hosts, therefore, encompasses two complementary strategies: modifying carbon surface chemistry to improve compatibility with sulfur species and SE particles, and constructing continuous conductive networks that maintain electronic connectivity under mechanical stress. These approaches focus on the chemical and mechanical functionality of carbon frameworks at the composite level rather than sulfur confinement alone. Detailed interfacial reaction mechanisms and the formation of chemically distinct interphases are discussed separately in Section 4.2.

Heteroatom doping illustrates how carbon surface chemistry can be tuned to improve compatibility with sulfur species and SE particles. Sun et al. combined this concept with surface-accessible pore engineering by developing polyacrylonitrile-derived porous carbon fibers (PPCFs) with a dense conductive core and a N-doped microporous surface shell [70]. This design localized sulfur-hosting micropores on the fiber surface, allowing sulfur to contact both the carbon framework and LPSCl. The N-doped surface shell provided accessible polar sites, while the dense core maintained continuous electronic conduction, leading to improved capacity retention during extended cycling [70]. Extending this concept, Li et al. introduced metal–N sites into carbon hosts, which provided additional Li^+^ coordination environments and promoted hybrid ionic/electronic reaction boundaries, thereby improving sulfur utilization and SE contact stability [71].

Surface coating of the conductive network further expands this strategy by combining SE compatibility with mechanical compliance. Ma et al. coated hollow N-doped carbon nanotubes (NCNT) with polypyrrole (PPy) to form a PPy@NCNT composite conductor [61]. The PPy@NCNT framework provided a continuous electrode-level conductive network, while the viscoelastic PPy coating improved compatibility with sulfide SEs and buffered sulfur volume changes (Figure 2D) [61]. Compared with acetylene-black-based cathodes, the PPy@NCNT-containing cathode showed a nearly one-order-of-magnitude higher effective electronic conductivity of 1.23 × 10^−1^ S cm^−1^ and an 11% reduction in axial pressure at a sulfur loading of 3 mg cm^−2^. It further achieved an areal capacity of 6 mAh cm^−2^ at 4.5 mg cm^−2^ sulfur loading and stable cycling for 300 cycles at 0.2 C [61].

A more mechanistically explicit example was reported by Meng et al., who used a carbon nitride/N-doped graphene host to suppress the oxidative decomposition of argyrodite LPSCl in ASSLSBs [72]. In this design, the N-rich carbon surface interacts strongly with Li^+^ in the argyrodite lattice, kinetically inhibiting Li^+^ extraction, which is regarded as an initial step in sulfide SE oxidation. As a result, the effective decomposition onset of LPSCl shifted to higher potentials, improving cathode–SE stability under operating conditions. This study provides an important design principle: carbon hosts in ASSLSB cathodes should be engineered not only for electronic conductivity, but also for chemical compatibility with adjacent SE particles [72].

Overall, surface- and network-engineered carbon cathodes expand the function of carbon from a passive electronic additive to a chemically and mechanically active component of the composite cathode. These strategies improve sulfur accessibility, SE compatibility, electronic connectivity, and contact retention during repeated S/Li_2_S conversion. The guiding design principle is that the carbon host must be engineered for dual compatibility—polar surface chemistry to anchor sulfur/Li_2_S species and controlled reactivity toward adjacent SE particles to suppress carbon-accelerated SE oxidation—rather than for electronic conductivity alone. The following section turns from carbon-host engineering within S/C composites to alternative cathode chemistries that modify the active material or redox environment itself.

### 2.2. Alternative Composite Cathodes

In addition to elemental sulfur dispersed in carbon hosts, ASSLSBs have also been developed using alternative composite cathode chemistries and functional cathode architectures. These approaches modify the active material, host phase, or redox environment to improve charge transport, sulfur utilization, mechanical stability, or reaction kinetics under solid-state conditions. Representative examples include Li_2_S-based cathodes, which start from the fully lithiated sulfur species; metal sulfide cathodes, which introduce electronically conductive and redox-active host phases; and other functional composite cathodes incorporating organosulfur materials, or redox-active additives.

#### 2.2.1. Li_2_S-Based Cathodes

As introduced in Section 1.3.1, Li_2_S avoids the initial volume expansion of S_8_ and is compatible with lithium-free anode configurations. However, its poor electronic and ionic conductivity (~10^−13^ and ~10^−9^ S cm^−1^, respectively) and high activation barrier on initial charge (>3 V vs. Li^+^/Li) require targeted design strategies to achieve practical utilization [73,74].

**Figure 3:**
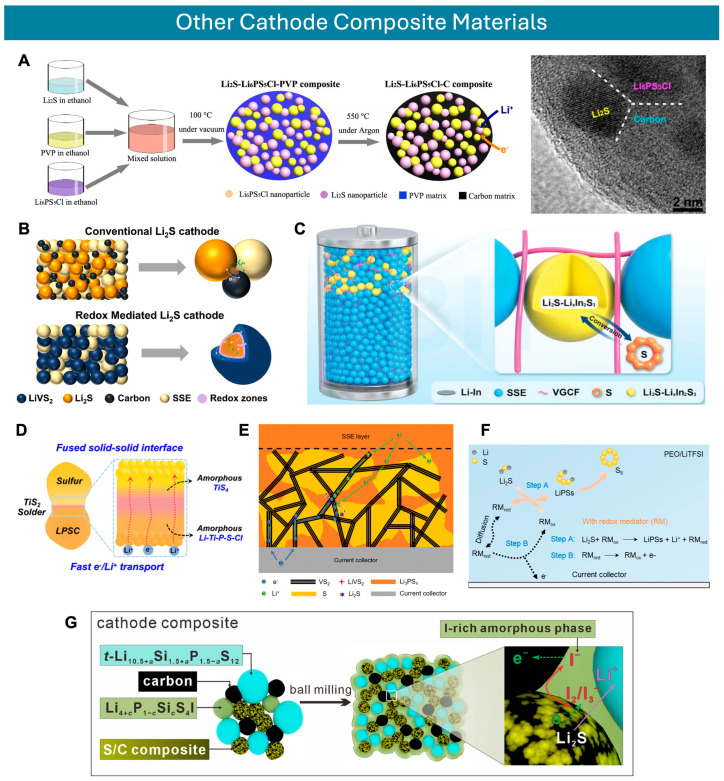
Alternative cathode composite strategies for ASSLSBs. (**A**) Solution-derived bottom-up synthesis of Li_2_S/LPSCl/C nanocomposite cathode with nanoscale triple-phase contact among Li_2_S, LPSCl, and carbon, as confirmed by TEM (Reproduced with permission from Ref. [74], copyright 2016 American Chemical Society). (**B**) LiVS_2_-coated Li_2_S cathode, in which the mixed-conducting LiVS_2_ shell acts as a solid-state redox mediator and expands the active reaction zone beyond conventional point contacts (Reproduced with permission from Ref. [73], copyright 2023 Royal Society of Chemistry). (**C**) Li_2_S–Li_x_In_2_S_3_ composite cathode for improved charge transport and volume-change mitigation (Reproduced with permission from Ref. [77], copyright 2023 Wiley-VCH). (**D**) TiS_2_-assisted interfacial soldering strategy, where amorphous TiS_4_ and Li–Ti–P–S–Cl phases fuse S/LPSCl solid–solid interfaces and promote coupled e^−^/Li^+^ transport (Reproduced with permission from Ref. [81], copyright 2025 Wiley-VCH). (**E**) S/VS_2_ hybrid cathode integrating sulfur conversion with VS_2_-assisted transport (Reproduced with permission from Ref. [82], copyright 2020 Wiley-VCH). (**F**) Molecular redox mediator for promoting Li_2_S oxidation in polymer-based solid-state cells (Reproduced with permission from Ref. [83], copyright 2021 American Chemical Society). (**G**) Iodine-rich sulfide catholyte enabling I^−^/I_2_/I_3_^−^ redox mediation at Li_2_S interfaces (Reproduced from Ref. [84]).

The foundational strategy to address the conductivity problem is the mixed-conducting nanocomposite, in which Li_2_S, SE, and carbon are co-distributed at the nanoscale to establish percolating ionic and electronic pathways. Han et al. demonstrated this through a bottom-up co-precipitation approach, yielding a Li_2_S–LPSCl–C composite with ionic and electronic conductivities far exceeding those measured for pristine Li_2_S, and achieving 830 mAh g^−1^ for 60 cycles at room temperature at a loading of 3.6 mg cm^−2^ (Figure 3A) [74]. Subsequent work extended this concept to in situ-generated Li_2_S–C composites at higher areal loadings [75] and to cathode-supported cell architectures with thin SE layers that raise cell-level energy density [76].

Addressing the activation barrier requires expanding the accessible reaction zone beyond the conventional triple-phase boundary. Kwok et al. encapsulated Li_2_S with an approximately 30 nm LiVS_2_ shell that served simultaneously as a mixed ionic/electronic conductor and a solid-state redox mediator (Figure 3B) [73]. During charge, the shell was proposed to facilitate Li_2_S oxidation by mediating charge transfer at the Li_2_S/LiVS_2_ interface, thereby mitigating the nucleation barrier associated with direct Li_2_S oxidation. The resulting cathode delivered rate capability up to 3 mA cm^−2^ at room temperature and 77% capacity retention over 1000 cycles at 1 mA cm^−2^ [73]. A complementary materials-level strategy was demonstrated by Yu et al., who showed that compositing Li_2_S with In_2_S_3_ suppresses the relative volumetric change by ~70%, as confirmed by in situ transmission electron microscopy (TEM), while Li_x_In_2_S_3_ simultaneously improves charge-carrier transport and mediates Li_2_S oxidation kinetics (Figure 3C) [77].

Additional approaches to Li_2_S cathode engineering include in situ formation of Li_3_PS_4_ at the Li_2_S particle surface to reduce interfacial resistance [78], CuS/N-doped carbon hosts that improve mixed-conducting transport around the active material [79], and the use of oxidation-tolerant SEs to raise Li_2_S utilization without modifying the active material itself [80]. These studies collectively show that Li_2_S-based cathodes are a promising alternative active-material platform for ASSLSBs, but their practical implementation requires integrated control of Li_2_S activation, mixed ion/electron transport, and chemo-mechanical stability. As a design principle, effective Li_2_S utilization requires the activation pathway, mixed ionic/electronic transport, and chemo-mechanical stability to be engineered together rather than optimized in isolation.

#### 2.2.2. Metal Sulfide Cathodes

Transition metal sulfides have been examined as functional cathode components that can mitigate the intrinsically low electronic conductivity and slow solid-state reaction kinetics of elemental sulfur. Compared with insulating S_8_/Li_2_S, many metal sulfides provide higher electronic conductivity, redox activity, and, in some cases, Li^+^ transport pathways [85,86]. They can therefore serve not only as conductive hosts but also as active or mediating phases that participate in conversion, intercalation, or hybrid sulfur redox processes. However, their higher molar mass reduces the gravimetric advantage of sulfur, making hybrid architectures that combine a limited amount of metal sulfide with a high sulfur payload particularly attractive [87].

Various metal sulfide cathodes and sulfur–metal sulfide hybrids have been examined in ASSLSBs. CuS-based cathodes were among the early examples, benefiting from high electronic conductivity but also showing limitations associated with displacement reactions and Cu redistribution under solid-state conditions [85,86]. FeS_2_ has been used as a sulfophilic and redox-active component in S/FeS_2_ hybrid cathodes, improving sulfur utilization and cycling stability relative to bare FeS_2_ [87]. TiS_2_ illustrates how a single metal sulfide can serve different functions depending on its design context. In Li_2_S@TiS_2_ core–shell cathodes, the TiS_2_ shell confined polysulfide species and promoted Li_2_S oxidation in polymer SE cells (~910 mAh g^−1^, 427 Wh kg^−1^ at 4.0 mg cm^−2^) [88]. Li et al. further demonstrated TiS_2_ as a catalytic “solder”: at just 3 wt%, it reacted during ball milling with both S_8_ and LPSCl to generate amorphous TiS_4_ together with mixed Li-Ti-P-S-Cl interfacial phases that fused the solid–solid contacts within the cathode, lowering the S_8_-to-Li_2_S activation energy from 18.2 to 8.6 kJ mol^−1^ and enabling 720 mAh g^−1^ after 2000 cycles at 1 C and 7.05 mAh cm^−2^ at 4.0 mg cm^−2^ (Figure 3D) [81].

A representative demonstration of the hybrid metal sulfide concept was reported by Xu et al., who used layered VS_2_ as a mixed-conducting and redox-active host for sulfur (Figure 3E) [82]. Elemental sulfur was melt-diffused into VS_2_ nanoplate aggregates to form an S/VS_2_ quasi-core–shell composite, which was then blended with β-Li_3_PS_4_. In this architecture, sulfur coating on the VS_2_ surface helped reduce direct contact between metallic VS_2_ and the thiophosphate SE, thereby limiting electrolyte oxidation during charge. Ex situ XRD confirmed a simultaneous conversion–intercalation mechanism, in which sulfur underwent S/Li_2_S conversion while VS_2_ reversibly lithiated to LiVS_2_; the partially lithiated Li_x_VS_2_ phase was proposed to assist Li^+^/electron delivery to sulfur. As a result, the S/VS_2_/Li_3_PS_4_ cathode achieved approximately 85% sulfur utilization, nearly 100% Coulombic efficiency after activation, and a high areal capacity of 7.8 mAh cm^−2^ at an active-material loading of 15.5 mg cm^−2^ [82].

Beyond VS_2_, other metal sulfide systems, including MoS_2_, CoS_2_, and SnS, and related sulfides, have also been investigated to tune electronic conductivity, Li^+^ transport, active surface area, and conversion reversibility in solid-state sulfur cathodes [89,90,91,92,93,94]. To summarize, metal sulfide cathodes introduce additional design routes of ASSLSBs by coupling sulfur conversion with electronically conductive and redox-active host phases. The central design challenge for metal sulfide cathodes is balancing the conductivity and kinetic benefits of the metal sulfide phase against its added mass and interfacial compatibility with SEs. This favors hybrid architectures that pair a minor redox-active metal-sulfide fraction with a high sulfur payload.

#### 2.2.3. Other Functional Composite Cathodes

This section covers cathode designs in which sulfur is either chemically incorporated into a polymer backbone or combined with small-molecule redox-active additives that mediate S/Li_2_S conversion. Both approaches aim to improve solid-state sulfur redox kinetics and SE compatibility by modifying the local chemical environment of sulfur, while maintaining a high sulfur contribution to the cathode capacity.

Sulfurized polyacrylonitrile (SPAN) is among the most mature organosulfur cathode materials for ASSLSBs. Prepared by co-pyrolysis of sulfur and polyacrylonitrile (PAN) at 280–450 °C under N_2_, SPAN covalently incorporates short sulfur chains into a nitrogen-rich conjugated carbon framework [95]. Because sulfur is chemically bound within the polymer-derived matrix, sulfur redox proceeds through a more localized pathway with minimal formation of soluble polysulfide intermediates, leading to high Coulombic efficiency and improved compatibility with solid-state configurations. Wang et al. assembled ASSLSBs using a SPAN/C cathode with a sandwich composite electrolyte, delivering 1793 mAh g^−1^ at 75 °C with stable cycling over 120 cycles [96]. Chalcogen doping further improves SPAN performance: Se doping enhanced Li^+^ diffusivity and electronic conductivity, giving 654 mAh g^−1^ with 81% retention over 500 cycles [97]. In addition, tellurium-substituted Te_0.05_S_0.95_@pPAN, when paired with an in situ-formed Li_7_P_3_S_11_ coating, achieved cathode–electrolyte interfacial compatibility sufficient for stable cycling beyond 500 cycles at room temperature [98]. Beyond SPAN, inverse vulcanization of sulfur with divinylbenzene (DVB) produces p(S-DVB) copolymer cathodes, in which C–S_n_–C linkages chemically confine sulfur species; the assembled ASSLSB exceeded 1100 mAh g^−1^ in the first cycle and retained 650 mAh g^−1^ after 50 cycles with a polymer SE at 70 °C [99]. Copolymerization with poly(ethylene glycol) methyl ether methacrylate (PMEMA) and crosslinking with 1,3-diisopropenylbenzene (DIB) further yielded poly(S-PMEMA-DIB), combining chemical sulfur confinement with improved dispersibility and SE compatibility in all-solid-state cells [100].

An alternative strategy introduces small redox-active molecules into the composite cathode to lower the kinetic barrier of solid-state S/Li_2_S conversion. Gao et al. incorporated an anthraquinone-based mediator (AQT) into a Li_2_S cathode composite with a PEO/lithium bis(trifluoromethanesulfonyl)imide (LiTFSI) SE [83]. AQT satisfies two important requirements for a solid-state mediator: a redox potential slightly above that of Li_2_S, enabling mediated charge transfer during Li_2_S oxidation, and sufficient compatibility within the polymer electrolyte matrix to maintain contact with the active material (Figure 3F) [83]. The Li_2_S/AQT cathode retained 88% capacity after 20 cycles at 60 °C, demonstrating that organic redox mediators originally developed for liquid cells can be adapted to polymer-based solid-state systems [83].

Iodine-containing sulfide catholytes represent a more recent direction for redox-mediated sulfur conversion in ASSLSBs. Yang et al. designed Li_10.5−x_Si_1.5_P_1.5_S_12−x_I_x_ catholytes, where iodine substitution generated an iodine-rich amorphous phase together with superionic nanocrystalline domains (Figure 3G) [84]. The iodine-rich phase introduced reversible redox mediation: iodine was proposed to be oxidized at the carbon|SE interface, migrate to the SE|Li_2_S interface, and chemically oxidize Li_2_S, thereby activating otherwise limited two-phase reaction sites. The optimized Li_10.1_Si_1.5_P_1.5_S_11.6_I_0.4_ catholyte delivered 1175 mAh g^−1^ at 5 C, and 590 mAh g^−1^ at 15 C [84].

Across both organosulfur and redox-mediator categories, the shared design principle is reducing the activation energy of the solid-state S/Li_2_S conversion—either by covalently constraining sulfur within a conductive polymer matrix that suppresses polysulfide formation entirely, or by introducing a molecular intermediary that bridges poorly contacted solid–solid interfaces.

## 3. Solid Electrolytes

The solid electrolyte is a defining component of ASSLSBs, as replacing the liquid phase suppresses polysulfide dissolution and mitigates the associated shuttle effect. In contrast to conventional liquid-electrolyte systems, the SE also replaces the separator and therefore must fulfill multiple roles within a single material, including fast Li^+^ transport, electronic insulation, mechanical robustness, and chemical compatibility with both electrodes. For practical ASSLSB operation, the SE should exhibit a room-temperature ionic conductivity approaching or exceeding 10^−3^ S cm^−1^, a Li^+^ transference number close to unity, and an electrochemical stability window compatible with sulfur redox reactions (typically ~1.5–2.8 V vs. Li/Li^+^). In addition, chemical and electrochemical stability against both sulfur cathodes and lithium metal anodes, along with adequate mechanical deformability and scalable processability, are required for long-term cell operation and practical manufacturing.

Despite extensive progress, no existing SE system simultaneously satisfies all of these requirements. Most ASSLSBs continue to suffer from low room-temperature ionic conductivity, large interfacial resistance arising from insufficient solid–solid contact, and sluggish solid-state sulfur redox kinetics. Moreover, the dominant limitations strongly depend on the composition, mechanical properties, and microstructure of the SE. Consequently, substantial research efforts have focused on improving Li^+^ transport, stabilizing electrode–electrolyte interfaces, and mitigating kinetic limitations across diverse SE material systems [21,101,102].

In this section, SEs are categorized into three major classes: inorganic solid electrolytes (Section 3.1), polymer electrolytes (Section 3.2), and hybrid composite electrolytes (Section 3.3). For each category, the ion-transport characteristics, key advantages, intrinsic limitations, and representative engineering strategies are discussed.

### 3.1. Inorganic Solid Electrolytes

Inorganic SEs are rigid crystalline or glassy materials in which Li^+^ ions migrate through fixed lattice frameworks via vacancy hopping or interstitial mechanisms. They offer high thermal stability and room-temperature ionic conductivities that in the best cases approach or exceed those of liquid electrolytes [27]. Their nonflammable character can reduce flammability-related safety risks associated with organic solvents, although interfacial instability with lithium metal remains a critical concern. The primary limitations are poor interfacial contact with composite electrodes due to mechanical rigidity, chemical instability against lithium metal or moisture, and demanding processing requirements. Comprehensive treatments of inorganic SE materials and their physicochemical properties are available in dedicated reviews [103,104,105]. This section focuses specifically on their application in ASSLSBs, with emphasis on how each SE class has been implemented in sulfur cell configurations and what electrochemical outcomes have been reported.

#### 3.1.1. Sulfide-Based Inorganic SEs

Sulfide SEs are among the most widely investigated inorganic SEs for ASSLSBs owing to their high room-temperature Li^+^ conductivity and favorable mechanical compliance. The high polarizability of S^2−^ weakens Li^+^–anion interactions, enabling fast Li^+^ migration, while the soft sulfide framework allows densification by cold pressing without high-temperature sintering [25,27]. Representative systems include β-Li_3_PS_4_, Li_2_S–P_2_S_5_ glasses and glass ceramics, LGPS, and argyrodite-type Li_6_PS_5_X (X = Cl, Br, I) [39,106,107,108]. However, their practical use is restricted by moisture instability with H_2_S evolution, limited oxidative stability, and interfacial reactivity with lithium metal. Accordingly, recent studies have pursued compositional and functional electrolyte designs to improve their conductivity, stability, and sulfur redox kinetics.

Among sulfide-based inorganic SEs, glass and glass-ceramic thiophosphate electrolytes have been widely investigated. Tufail et al. reported an aliovalent dual-doped Li_7_P_3_S_11_-based electrolyte, Li_7_Sb_0.05_P_2.95_S_10.5_I_0.5_, in which Sb^5+^ and I^−^ were introduced to improve both Li^+^ transport and moisture sensitivity [109]. Sb^5+^/I^−^ co-doping was proposed to enlarge Li^+^ migration channels, introduce structural defects, and lower the activation barrier, resulting in a room-temperature ionic conductivity of 2.55 × 10^−3^ S cm^−1^ and an activation energy of 20.04 kJ mol^−1^. The partial substitution of P^5+^ with softer Sb^5+^ also strengthened Sb–S interactions and reduced moisture-induced H_2_S evolution. When used in ASSLSBs with a Li_2_S–C–SE composite cathode, the doped electrolyte enabled an initial discharge capacity of 622.3 mAh g^−1^ and retained 687.3 mAh g^−1^ after 15 cycles at room temperature [109].

Beyond compositional modification to improve conductivity and stability, recent studies have expanded the role of sulfide SEs from passive ion conductors to functional components that directly mediate sulfur redox chemistry. Song et al. developed lithium thioborophosphate iodide (LBPSI) glass-phase electrolytes that mediate sulfur redox through reversible I^−^/I_2_/I_3_^−^ chemistry [110]. During charge, surface I^−^ species are oxidized to I_2_/I_3_^−^ at the carbon|SE interface and subsequently chemically oxidize Li_2_S at SE|Li_2_S two-phase boundaries, thereby increasing the number of accessible reaction sites beyond conventional triple-phase contacts. The optimized LBPSI electrolyte exhibited a room-temperature ionic conductivity of 2.4 mS cm^−1^ and enabled ASSLSBs to deliver 784 and 447 mAh g^−1^ at 20 C and 35 C, respectively, at 30 °C. The cell also retained 80.2% of its capacity after 25,000 cycles at 5 C [110].

These studies illustrate the evolution of sulfide SE design from conductivity- and stability-oriented compositional tuning toward functional electrolyte engineering that directly modulates sulfur redox kinetics.

#### 3.1.2. Oxide-Based Inorganic SEs

Oxide-based SEs represent another important class of inorganic electrolytes for ASSLSBs, offering superior chemical stability, moisture tolerance, and mechanical robustness compared with sulfide counterparts. Their strong metal–oxygen frameworks provide high oxidative stability and improved environmental compatibility, which are advantageous for practical handling and long-term cell operation [111]. Representative oxide SEs include garnet-type LLZO, Li_1+x_Al_x_Ge_2−x_(PO_4_)_3_ (LAGP), NASICON-type Li_1+x_Al_x_Ti_2−x_(PO_4_)_3_ (LATP) and perovskite-type Li_3x_La_2/3−x_TiO_3_ (LLTO), and anti-perovskite-type lithium oxyhalides [112,113,114]. However, oxide SEs generally suffer from higher grain-boundary resistance, lower deformability, and poor solid–solid interfacial contact with sulfur composite cathodes [25]. Their high sintering temperatures and mechanical rigidity also complicate scalable electrode fabrication. Accordingly, recent efforts have focused on reducing interfacial resistance, improving cathode–electrolyte contact, and developing composite or interlayer strategies to exploit the stability of oxide SEs in Li–S battery configurations.

Among oxide-based SEs, garnet-type LLZO derivatives have been widely examined for Li–S battery configurations because of their chemical stability, dense ceramic structure, and compatibility with lithium metal. Xu et al. demonstrated an all-in-one Li–S cell using a porous–dense–porous trilayer Nb-doped LLZO electrolyte, in which the dense middle layer suppressed lithium dendrite penetration and polysulfide crossover, while the porous outer layers served as hosts for lithium metal and sulfur [115]. This architecture provided continuous Li^+^ pathways and accommodated volume changes during cycling. Although a small amount of liquid electrolyte was still required at the cathode side to improve contact, the cell achieved ~1200 mAh g^−1^ and maintained nearly 100% Coulombic efficiency over 50 cycles [115].

Lu et al. further demonstrated a garnet-based Li–S cell using dense Ta-doped Li_6.4_La_3_Zr_1.4_Ta_0.6_O_12_ (LLZTO) with modified anodic and cathodic interfaces [116]. The dense LLZTO membrane acted as a physical barrier to polysulfide migration, while an Au coating on the anode side improved Li wetting and reduced Li/LLZTO interfacial resistance. On the cathode side, P_2_S_5_/Li_2_S additives in the liquid catholyte enhanced Li_2_S solubility and promoted formation of a Li_3_PS_4_-based Li^+^-conductive interphase. With these interface modifications, the LLZTO-based cell retained 805 mAh g^−1^ after 500 cycles and achieved high capacities of 1088 and 799 mAh g^−1^ at sulfur loadings of 3.2 and 5.3 mg cm^−2^, respectively [116].

Overall, garnet-type oxide SEs offer clear advantages as dense and chemically stable barriers for suppressing polysulfide crossover and lithium dendrite penetration. Nevertheless, their poor deformability and high solid–solid interfacial resistance remain major obstacles for ASSLSBs, often necessitating small amounts of liquid electrolyte or catholyte to improve cathode utilization. Future oxide-based Li–S cell designs should therefore focus on reducing cathode–electrolyte contact resistance and developing fully solid interfacial architectures that retain the intrinsic stability of oxide SEs while enabling efficient sulfur redox reactions.

#### 3.1.3. Halide-Based Inorganic SEs

Halide-based SEs have recently emerged as promising inorganic electrolytes for ASSLSBs because they combine relatively high Li^+^ conductivity, good deformability, and improved oxidative stability compared with sulfide SEs [117]. Their halogen-based anion frameworks can provide favorable Li^+^ migration pathways while enabling better processability and electrode contact than rigid oxide ceramics [35]. In particular, rare-earth-based halide electrolytes have attracted attention because their crystal structures contain partially occupied Li sites and vacant octahedral sites that facilitate Li^+^ migration. However, many halide SEs are thermodynamically unstable against lithium metal and can undergo reductive decomposition at the Li interface [118]. Therefore, buffer layers or interfacial protection strategies are often required when halide SEs are paired with Li metal anodes [119].

As a representative example, Shi et al. reported a rare-earth bromide electrolyte, Li_3_HoBr_6_ (LHB), for all-solid-state Li–S batteries [120]. The LHB electrolyte exhibited a room-temperature ionic conductivity of 1.1 × 10^−3^ S cm^−1^ and could be readily cold-pressed into compact pellets, reflecting its favorable deformability. Density functional theory (DFT) calculations identified multiple Li^+^ migration pathways in the LHB structure, among which out-of-plane migration through Br-based tetrahedral sites provided low migration barriers and contributed to fast Li^+^ transport. Compared with sulfide electrolytes, LHB showed a wider electrochemical stability window of 1.5–3.3 V vs. Li/Li^+^, which is compatible with sulfur redox chemistry. Nevertheless, because LHB can be reduced by Li metal, a Li_7_P_3_S_11_ buffer layer was introduced between the Li anode and LHB to suppress direct interfacial reactions. The resulting Li/Li_7_P_3_S_11_/LHB/S cell delivered a discharge capacity of 582 mAh g^−1^ at 0.1 C and maintained stable cycling for 400 cycles with nearly 100% Coulombic efficiency at 0.2 C and 60 °C [120].

Overall, halide SEs offer an attractive balance between the processability of sulfides and the oxidative stability of oxides, making them promising candidates for ASSLSBs. However, their reductive instability against lithium metal and the need for interfacial buffer layers remain important limitations. Stabilizing the Li/halide interface and improving room-temperature sulfur redox kinetics remain the primary obstacles for halide-based ASSLSBs.

#### 3.1.4. Hydride-Based Inorganic SEs

Hydride-based SEs, particularly complex metal hydrides containing borohydride anions such as BH_4_^−^, have also been explored as alternative inorganic electrolytes for ASSLSBs. These materials are attractive because of their relatively soft nature, favorable interfacial contact, and good compatibility with lithium metal [29]. However, their practical use is often limited by low room-temperature ionic conductivity, restricted electrochemical stability depending on composition, and possible side reactions associated with hydride species [121].

Das et al. demonstrated an ASSLSB using LiBH_4_ nanoconfined in mesoporous silica MCM-41 as the solid electrolyte [122]. Nanoconfinement stabilizes LiBH_4_ in a highly conductive state and improves Li^+^ transport compared with bulk LiBH_4_, yielding ionic conductivities of approximately 0.1 mS cm^−1^ at room temperature and 0.2 mS cm^−1^ at 55 °C. The electrolyte also exhibited negligible electronic conductivity, a high Li^+^ transference number of 0.96, and stable cycling in Li symmetric cells, supporting its compatibility with lithium metal. When applied to Li–S cells, the battery retained a capacity of approximately 1220 mAh g^−1^ after 40 cycles at 55 °C and 0.03 C, with an average discharge voltage of ~2 V. However, an anomalously high first-discharge capacity was attributed to parasitic reactions between LiBH_4_ and the sulfur cathode, likely involving cathode–electrolyte interphase formation, and the capacity decreased at higher C-rates because of polarization and incomplete sulfur utilization. These results demonstrate the potential of nanoconfined hydride electrolytes for Li–S chemistry, although further improvements in room-temperature conductivity, cathode compatibility, and rate capability are required [122].

These inorganic SE examples show that high Li^+^ conductivity alone is insufficient for ASSLSBs; mechanical compliance and interfacial compatibility are equally critical, motivating the polymer and hybrid electrolyte approaches discussed below.

### 3.2. Polymer Electrolytes

Unlike inorganic SEs, which conduct Li^+^ through fixed crystalline or glassy frameworks, polymer SEs rely primarily on segmental chain motion to facilitate Li^+^ transport. Li^+^ ions coordinate with electron-donating sites, most commonly ether oxygen groups in the polymer backbone, and migrate through intra- or interchain hopping coupled to local relaxation of amorphous polymer segments [123,124,125,126]. This transport mechanism gives polymer SEs a set of properties complementary to inorganic SEs, particularly mechanical flexibility and electrode conformability, but it also imposes intrinsic conductivity and stability limitations.

The principal advantages of polymer SEs in ASSLSBs are their mechanical compliance, processing versatility, and relatively favorable compatibility with lithium metal. Soft polymer matrices can deform against composite cathode and anode surfaces during cycling, helping to maintain interfacial contact and reduce solid–solid interfacial resistance without requiring the high stack pressures often needed for inorganic SEs [21]. Their scalable fabrication by solution casting, hot pressing, electrospinning, or in situ polymerization further supports electrode-integrated cell architectures. Among the polymer hosts studied, PEO is the most widely investigated because ether oxygen groups coordinate Li^+^ effectively, amorphous PEO segments support ion hopping, and its electrochemical stability is generally compatible with the voltage range of sulfur redox chemistry [21].

However, polymer SEs suffer from two interrelated limitations that constrain their application in ASSLSBs. First, the ionic conductivity of PEO-based electrolytes rarely exceeds 10^−4^ S cm^−1^ at room temperature because Li^+^ transport is coupled to segmental motion, which is strongly suppressed below the melting temperature of PEO [127]. As a result, many PEO-based ASSLSBs require operation at elevated temperatures, typically 60–80 °C, to achieve sufficient ionic conductivity and sulfur utilization [102]. Second, and more specific to Li–S chemistry, PEO and other polar polymer matrices can solvate lithium polysulfide intermediates when they form during cycling, allowing their migration from the cathode toward the anode and partially reintroducing shuttle-like behavior [9,21]. This polysulfide-solvation issue distinguishes polymer SE-based ASSLSBs from inorganic SE-based systems and necessitates cathode-side containment strategies in addition to ionic conductivity improvement.

Accordingly, polymer-based electrolytes for ASSLSBs range from fully solvent-free solid polymer electrolytes (SPEs) to gel polymer electrolytes (GPEs) that incorporate plasticizers or limited liquid phases to enhance ionic conductivity. The following discussion covers both types: SPEs (Section 3.2.1), which prioritize solvent-free operation, mechanical integrity, and stable electrode contact, and GPEs (Section 3.2.2), which incorporate plasticizers or limited liquid phases to improve ionic conductivity and cathode wetting at the cost of some safety margin.

#### 3.2.1. Solid Polymer Electrolytes

**Figure 4:**
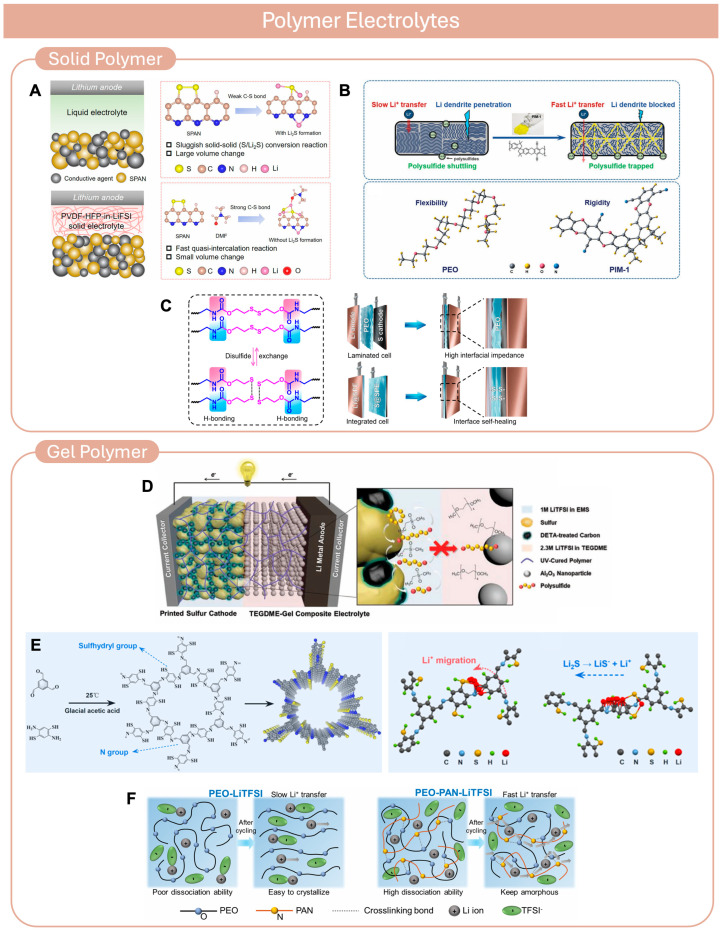
Polymer electrolyte for ASSLSBs. (**A**) PVDF-HFP-in-LiFSI solid polymer electrolyte enabling fast quasi-intercalation reactions and reduced volume change in SPAN-based solid-state cells (Reproduced with permission from Ref. [128], copyright 2022 Royal Society of Chemistry). (**B**) PEO–PIM composite electrolyte, where rigid PIM-1 suppresses polysulfide shuttling, blocks Li dendrite penetration, and enhances Li^+^ transport (Reproduced with permission from Ref. [129], copyright 2021 Wiley-VCH). (**C**) Dynamic poly(ether-urethane)-based solid polymer electrolyte with disulfide exchange and hydrogen bonding for interfacial self-healing (Reproduced from Ref. [130]). (**D**) TEGDME-based gel composite electrolyte combined with a printed sulfur cathode for improved interfacial contact and polysulfide confinement (Reproduced with permission from Ref. [134], copyright 2019 Wiley-VCH). (**E**) Sulfhydryl- and nitrogen-functionalized gel polymer electrolyte promoting Li^+^ migration and Li_2_S conversion (Reproduced from Ref. [135]). (**F**) Crosslinked PEO–PAN–LiTFSI polymer electrolyte with enhanced salt dissociation and suppressed crystallization (Reproduced with permission from Ref. [136], copyright 2022 Wiley-VCH).

Fully solvent-free SPEs have been developed to overcome the low room-temperature conductivity and interfacial instability of conventional PEO-based electrolytes. Li et al. reported a polymer-in-salt SPE based on a poly(vinylidenefluoride-co-hexafluoropropylene)-in-lithium bis(fluorosulfonyl)imide (PVDF-HFP-in-LiFSI) for solid-state Li–SPAN batteries (Figure 4A) [128]. The high salt content reduced polymer crystallinity and generated ion-cluster-assisted Li^+^ transport pathways, enabling room-temperature operation without high stack pressure. More importantly, residual solvent strengthened the C–S bonding in SPAN and promoted a quasi-intercalation-type reaction, avoiding complete S-to-Li_2_S conversion and thereby reducing both kinetic barriers and volume change [128].

Functional polymer frameworks have also been used to reinforce PEO-based SPEs. Ji et al. introduced a polymer of intrinsic microporosity, PIM-1, into PEO to form a PEO–PIM composite electrolyte (Figure 4B) [129]. The rigid microporous PIM-1 framework reduced PEO crystallinity, enhanced mechanical strength, and improved Li^+^ transport, while electrophilic 1,4-dicyanooxanthrene groups helped immobilize lithium polysulfides. As a result, the optimized PEO–PIM electrolyte delivered more than 600 mAh g^−1^ at 2 C and retained 730 mAh g^−1^ after 100 cycles at 0.5 C and 60 °C, outperforming the pristine PEO electrolyte. This design simultaneously addressed conductivity, mechanical stability, and polysulfide migration in PEO-based Li–S cells [129].

Interfacial adaptability was further addressed by Pei et al., who developed a poly(ether-urethane)-based SPE containing dynamic disulfide bonds and hydrogen-bonding urethane groups (Figure 4C) [130]. The dynamic bonding network enabled interfacial self-healing and maintained intimate electrode/electrolyte contact during cycling. Using integrated electrode/electrolyte structures, Li/Li symmetric cells cycled for more than 6000 h, and solid-state Li–S cells based on SPAN cathodes operated for 700 cycles at 0.3 C [130]. In addition to these representative examples, diverse SPE designs based on polymer-in-salt formulations, crosslinked or block-copolymer matrices, single-ion conducting polymers, and functional polymer frameworks have been explored in solid-state Li–S cells [131,132,133].

#### 3.2.2. Gel Polymer Electrolytes

Compared with solvent-free SPEs, GPEs and quasi-solid polymer electrolytes incorporate plasticizers or limited liquid components to improve ionic conductivity and interfacial wetting. Kim et al. verified this concept in a printed bipolar ASSLSB by combining two nonflammable gel electrolytes that remain thermodynamically immiscible at the operating salt concentration (Figure 4D) [134]. The ethyl methyl sulfone (EMS)-based gel phase within the cathode improved local ion transport and contact with sulfur species, whereas the tetraethylene glycol dimethyl ether (TEGDME)-based gel composite electrolyte functioned as a polysulfide-blocking separator membrane. The immiscibility between the two gel phases helped suppress polysulfide crossover while enabling solvent-drying-free, UV-curing-assisted stepwise printing of flexible bipolar cells [134].

Functional GPE membranes have also been designed to regulate polysulfide chemistry under lean-electrolyte conditions. Bi et al. constructed a sulfhydryl-functionalized COF@PVDF-HFP quasi-solid electrolyte, in which COF-SH was grown in situ on polarized PVDF-HFP fibers (Figure 4E) [135]. The sulfhydryl and imine groups in the COF layer provided strong polysulfide adsorption and promoted catalytic conversion, enabling efficient Li^+^ conduction with reduced liquid electrolyte content. The resulting Li–S cells delivered 688.7 mAh g^−1^ at 2 C and retained 568.8 mAh g^−1^ after 800 cycles, demonstrating the effectiveness of chemically functionalized GPEs for suppressing shuttle behavior while maintaining reaction kinetics [135].

In a related GPE design by Sheng et al., a PEO-PAN (poly(ethylene oxide)–polyacrylonitrile) network was prepared in which the PAN nanofibers played a dual structural role—both reinforcing the membrane and providing crosslinking sites for the surrounding PEO matrix (Figure 4F) [136]. The resulting network simultaneously raised ionic conductivity, mechanical strength, and dendrite tolerance, while C=N–O moieties generated through PEO–PAN crosslinking served as polysulfide-anchoring sites. Owing to its flexibility and interfacial bonding ability, the resulting flexible Li–S cell retained more than 96% of its capacity after 1000 bending cycles, highlighting the importance of coupling ion transport, polysulfide suppression, and mechanical robustness in GPE design [136].

Beyond these representative examples, GPEs based on PEO, PVDF-HFP, PAN, ionic-liquid-containing gels, and ether- or sulfone-plasticized networks have been widely investigated to balance Li^+^ transport, polysulfide suppression, electrode conformability, and mechanical durability in quasi-solid-state Li–S batteries [137,138,139,140,141,142].

Overall, polymer electrolytes provide interfacial conformity and processability that are difficult to achieve with rigid inorganic SEs. SPEs emphasize solvent-free safety, mechanical integrity, and stable electrode contact, whereas GPEs improve ionic conductivity and cathode wetting through controlled plasticization or limited liquid uptake. Nevertheless, both classes must balance Li^+^ transport, polysulfide immobilization, Li metal stability, and mechanical durability. Future polymer electrolyte development should therefore focus on integrated molecular and interfacial design: decoupling Li^+^ transport from polymer segmental motion, suppressing polysulfide migration through functional polymer chemistry, maintaining adaptive electrode contact during cycling, and stabilizing the Li metal interface. Room-temperature operation and high sulfur loading under lean-electrolyte conditions remain the key benchmarks for polymer electrolyte development in ASSLSBs.

### 3.3. Hybrid Composite Electrolytes

Hybrid composite electrolytes refer to polymer-based electrolyte systems that incorporate inorganic components, such as ceramic fillers, sulfide or oxide particles, nanosheets, nanowires, or other ion-conducting phases. This strategy has been widely explored to combine the complementary advantages of polymer and inorganic SEs while mitigating their individual limitations [143,144,145]. In general, the polymer matrix provides mechanical flexibility, processability, and conformal electrode contact, whereas the inorganic component contributes higher ionic conductivity, mechanical reinforcement, thermal stability, and improved resistance to lithium dendrite growth [145,146].

Among various hybrid designs, dispersing ceramic fillers or structured inorganic phases within a polymer matrix is the most common approach. These inorganic components can reduce polymer crystallinity, promote lithium salt dissociation, and create additional Li^+^ transport pathways at polymer–filler interfaces [147]. At the same time, rigid fillers reinforce the polymer matrix and improve dimensional stability, which is particularly important for maintaining electrode–electrolyte contact and stabilizing Li metal interfaces during cycling [148].

For ASSLSBs, hybrid electrolytes are especially attractive because they can simultaneously address ion transport, interfacial contact, mechanical durability, and polysulfide regulation. Recent studies have therefore moved beyond simple particle addition toward more structured and functional designs, including ceramic-decorated cathode/electrolyte frameworks, inorganic-rich polymer composites, laminar nanosheet electrolytes, thermally conductive fillers, ordered MOF-based transport regulators, and scalable ultrathin composite membranes [143,144,145,146,149,150].

**Figure 5:**
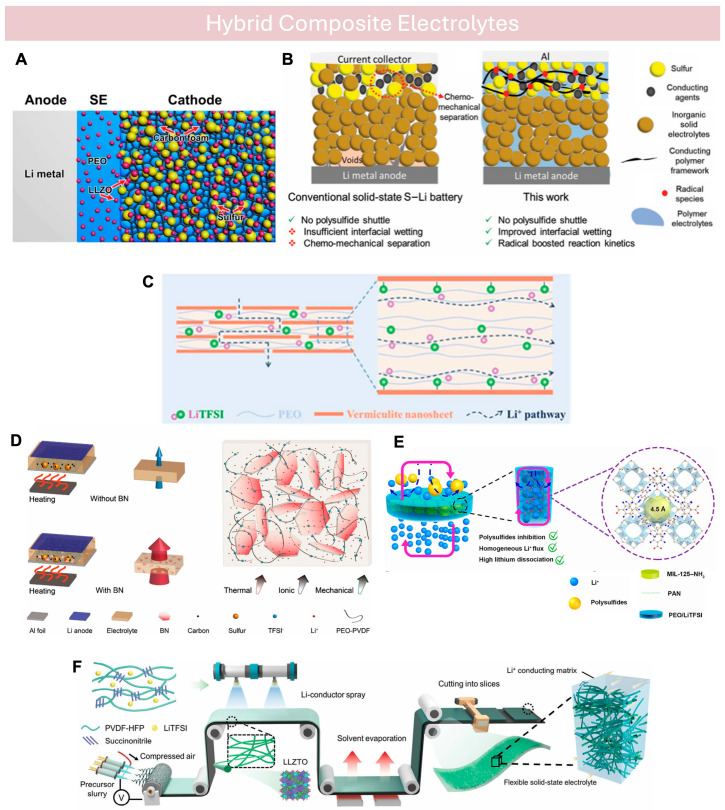
Hybrid composite electrolytes for ASSLSBs. (**A**) LLZO–PEO hybrid electrolyte coupled with a sulfur/carbon foam cathode for continuous interfacial Li^+^ transport across the cathode/electrolyte interface (Reproduced with permission from Ref. [143], copyright 2017 American Chemical Society). (**B**) Conducting polymer–inorganic electrolyte framework for improved wetting and reduced chemo-mechanical separation (Reproduced from Ref. [144]). (**C**) Vermiculite nanosheet/PEO composite electrolyte with ordered Li^+^ transport pathways (Reproduced with permission from Ref. [145], copyright 2020 Royal Society of Chemistry). (**D**) BN-containing composite polymer electrolyte for enhancing thermal, ionic, and mechanical stability (Reproduced from Ref. [149]). (**E**) MIL-125–NH_2_-modified PEO/PAN/LiTFSI electrolyte for polysulfide inhibition and homogeneous Li^+^ flux (Reproduced from Ref. [150]). (**F**) Roll-to-roll fabrication of an ultrathin LLZTO-reinforced composite polymer electrolyte (Reproduced with permission from Ref. [146], copyright 2024 Wiley-VCH).

Among early demonstrations of hybrid electrolytes for solid-state Li–S chemistry, Tao et al. embedded Al/Nb-codoped LLZO into both phases of the cell: dispersed within a PEO matrix as the electrolyte membrane, and grafted onto a porous carbon-foam sulfur host (Figure 5A) [143]. The key design principle was to provide Li^+^-conducting ceramic domains not only in the electrolyte membrane but also within the sulfur cathode, thereby reducing the discontinuity between cathode ion transport and bulk electrolyte transport. The LLZO filler improved Li^+^ transport in the polymer electrolyte, while the LLZO@C framework supplied coupled ionic/electronic pathways and enhanced sulfur utilization in the cathode. This hybrid configuration enabled relatively low-temperature operation for a PEO-based Li–S cell, delivering more than 900 mAh g^−1^ at 37 °C and higher capacities of 1210 and 1556 mAh g^−1^ at 50 and 70 °C, respectively [143].

Sulfide–polymer hybrid electrolytes provide another route to combining high ionic conductivity with improved interfacial contact. Li et al. reported a thio-LiSICON/polymer composite electrolyte by blending LGPS with plasticized PEO for solid-state Li–S batteries using a sulfurized polyacrylonitrile cathode (Figure 5B) [144]. The sulfide phase provided fast Li^+^ conduction, while the polymer component improved wettability and reduced interfacial resistance with the electrodes. The assembled cell delivered 1183 mAh g^−1^ at 0.2 C and 719 mAh g^−1^ at 0.5 C, showing that inorganic-rich hybrid electrolytes can support fast sulfur redox kinetics when paired with compatible organosulfur cathodes [144].

Beyond particle-type ceramic fillers, two-dimensional inorganic frameworks have been used to construct more continuous and mechanically robust Li^+^ transport pathways. By infiltrating PEO–LiTFSI into the interlayer galleries of stacked vermiculite nanosheets, Zhai et al. produced a brick-and-mortar–type thin-film composite electrolyte in which the inorganic layers acted as oriented Li^+^-transport channels (Figure 5C) [145]. This design created continuous interlayer Li^+^ transport channels with maximized polymer–inorganic interfacial contact, while the nanosheet architecture mechanically reinforced the electrolyte. The resulting thin electrolyte showed a low area-specific resistance of 66 Ω cm^2^ and improved Li–S cell rate performance, highlighting the value of ordered two-dimensional filler architectures over randomly dispersed particles [145].

Yin et al. introduced a thermal-management filler strategy by incorporating electrically insulating two-dimensional boron nitride (BN) nanoflakes into a PEO–PVDF/LiTFSI polymer electrolyte (Figure 5D) [149]. Unlike conventional fillers that mainly reduce polymer crystallinity or reinforce the matrix, BN was selected to improve heat transport through the electrolyte. This is important because Li^+^ conductivity in PEO-based electrolytes is strongly temperature-dependent, and local thermal gradients can cause nonuniform Li deposition and uneven sulfur conversion. The BN filler improved thermal uniformity, ionic conductivity, and mechanical strength, enabling more stable Li symmetric-cell cycling and improved ASSLSB performance compared with the filler-free polymer electrolyte [149].

More recently, Li et al. developed an ordered metal–organic framework (MOF) transport-regulation strategy using MIL-125–NH_2_ as a functional filler in a PEO-based composite solid electrolyte (Figure 5E) [150]. The ordered MOF framework was designed to simultaneously promote Li^+^ transport and suppress polysulfide migration: MIL-125–NH_2_ promoted LiTFSI dissociation and provided continuous Li^+^ transport channels, while its pore structure acted as a size-selective barrier against lithium polysulfides. This design directly addresses a key dilemma in polymer-based Li–S electrolytes—enhancing ion conductivity without increasing polysulfide mobility. The resulting electrolyte achieved an ionic conductivity of 8.3 × 10^−4^ S cm^−1^ at 60 °C, and pouch-type ASSLSBs maintained stable operation over 400 cycles under 0.5 C [150].

Scalable fabrication was further emphasized by Nie et al., who reported a large-scale ultrathin reinforced composite polymer electrolyte strategy based on electro-blown spinning (Figure 5F) [146]. In this design, a PVDF/polyurethane (PU)/LLZTO nanofiber skeleton was prepared as a mechanically robust porous support and then infiltrated with a PVDF-HFP/succinonitrile/LiTFSI electrolyte phase. The three-dimensional skeleton reinforced the thin membrane, restricted anion migration, and homogenized Li^+^ flux, thereby improving dendrite resistance and Li metal compatibility. The resulting electrolyte was approximately 18 μm thick, exhibited a room-temperature ionic conductivity of 1.0 × 10^−3^ S cm^−1^, and enabled the Li|SPAN cell to retain 94.0% capacity after 350 cycles at 0.5 C [146].

Overall, hybrid composite electrolytes broaden the range of viable design strategies of ASSLSBs by combining the high ionic conductivity, mechanical robustness, and thermal stability of inorganic SEs with the flexibility, processability, and interfacial conformity of polymer electrolytes. However, their performance is highly dependent on the dispersion, connectivity, and functionality of Li^+^-conducting components within the polymer matrix. Poorly designed hybrid systems may simply combine the drawbacks of both electrolyte classes, including limited ion transport, polysulfide migration, and interfacial instability. Future hybrid electrolyte design should therefore focus on constructing continuous Li^+^ transport networks and chemically compatible polymer–inorganic interfaces that simultaneously enhance ionic conductivity, suppress polysulfide shuttle, and stabilize electrode–electrolyte contact.

## 4. Interfaces

Interfaces play a decisive role in determining the electrochemical performance and durability of ASSLSBs. Although replacing liquid electrolytes with solid electrolytes suppresses polysulfide dissolution and improves safety, it also eliminates the liquid phase that would otherwise wet porous electrodes and maintain continuous ionic contact. As a result, sulfur redox reactions in ASSLSBs are confined to solid–solid contact regions where the active material, solid electrolyte, and electronic conductor are simultaneously connected. Any loss of contact at these interfaces directly interrupts Li^+^ and electron transport, increases polarization, and reduces active material utilization.

The interfacial challenges in ASSLSBs can be broadly divided into physical and chemical origins. Physical interfacial failure arises from insufficient wetting between rigid solids, nonuniform particle contact, interfacial void formation, and contact loss induced by the large volume change between sulfur and Li_2_S. These effects are particularly severe in composite cathodes, where repeated lithiation and delithiation can disrupt the percolated Li^+^/electron transport network. Chemical interfacial failure, in contrast, originates from the thermodynamic or electrochemical instability of solid electrolytes against lithium metal, sulfur species, carbon additives, or high interfacial potentials. The resulting decomposition products and interphases may be ionically resistive, electronically conductive, or mechanically unstable, thereby accelerating impedance growth and capacity decay.

In practice, these physical and chemical failure modes are not independent but are dynamically coupled in a self-reinforcing loop. Volume-change-induced contact loss exposes fresh, unpassivated SE surfaces and concentrates the local current density at the shrinking contact area, both of which accelerate electrochemical SE decomposition. The resulting interphases are often ionically resistive and mechanically brittle, and their formation introduces additional local stress that further promotes crack propagation and contact separation. Conversely, growth of a resistive interphase raises the local overpotential and drives inhomogeneous Li^+^/electron flux, intensifying the mechanical stress that aggravates physical contact loss. This closed-loop interplay—in which mechanical decohesion accelerates chemical degradation, and chemical degradation in turn aggravates mechanical failure—is a central reason why interfacial resistance in ASSLSBs grows progressively upon cycling and is rarely resolved by addressing either origin in isolation; physical contact and interfacial chemistry must instead be stabilized together.

Accordingly, interface engineering has become a central strategy for advancing ASSLSBs beyond material-level optimization of cathodes and solid electrolytes. In this section, recent interfacial strategies are discussed in two categories. Section 4.1 focuses on physical interface engineering, including pressure-assisted contact, mechanically compliant interlayers, and three-dimensional architectures that improve solid–solid contact and accommodate volume change. Section 4.2 discusses chemical interface engineering at cathode–SE and Li metal–SE interfaces, as well as in situ interphase formation strategies that suppress parasitic reactions and regulate interphase formation.

### 4.1. Physical Interface Engineering

In ASSLSBs, repeated sulfur/Li_2_S conversion can generate cracks, voids, and interfacial delamination, reducing the effective reaction area and disrupting percolated Li^+^/electron transport pathways. Accordingly, physical interface engineering aims to preserve continuous ion/electron transport pathways by controlling electrode–electrolyte morphology, mechanical compliance, and cell pressure.

**Figure 6:**
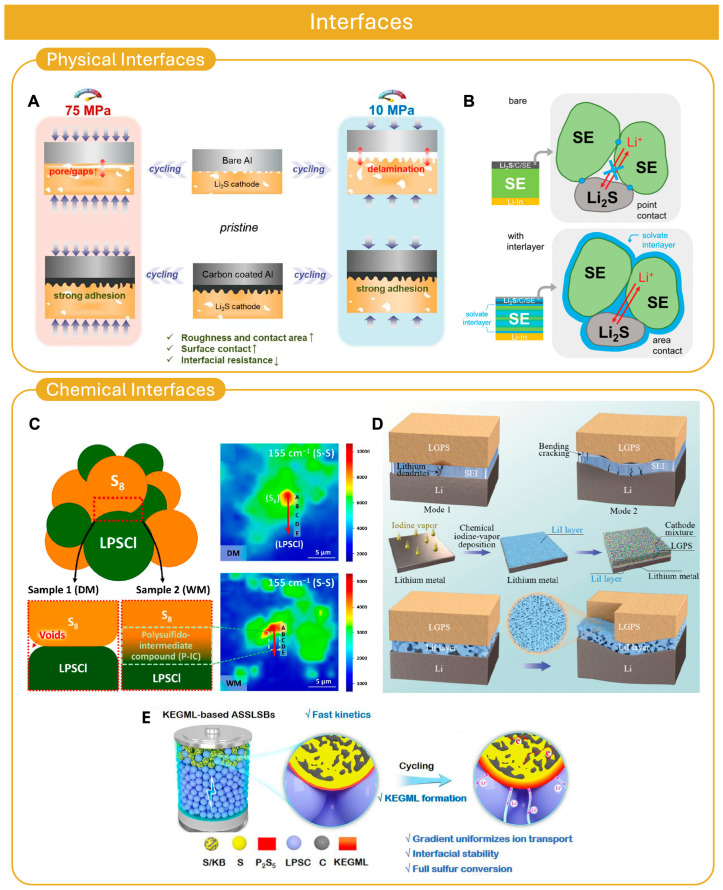
Physical and chemical interface engineering strategies for ASSLSBs. (**A**) Carbon-coated Al current collector for improving cathode/current-collector adhesion, increasing contact area, and reducing interfacial resistance under stack pressure (Reproduced from Ref. [152]). (**B**) Solvate interlayer strategy for converting point contacts into area contacts at Li_2_S/solid electrolyte interfaces (Reproduced with permission from Ref. [153], copyright 2019 Wiley-VCH). (**C**) Wet-mixing-derived cathode interface containing polysulfido-intermediate compounds to improve S_8_/LPSCl contact and sulfur redox kinetics (Reproduced from Ref. [155]). (**D**) Iodine-vapor-induced LiI interphase at the Li/LGPS interface for suppressing lithium dendrite propagation and stabilizing Li metal cycling (Reproduced with permission from Ref. [156], copyright 2022 Royal Society of Chemistry). (**E**) Kinetically enhanced gradient mixed layer (KEGML) formed during cycling to homogenize Li^+^ transport, stabilize the cathode interface, and promote full sulfur conversion (Reproduced from Ref. [157]).

Stack pressure provides a direct means of improving solid–solid contact in ASSLSBs. Lee et al. showed that increasing stack pressure in Li–In|LPSCl|Li_2_S cells enhanced active material utilization and capacity retention by preserving Li^+^/electron transport pathways and suppressing contact loss within the composite cathode [151]. However, reliance on high external pressure is impractical for scaled cell formats. Park et al. therefore proposed a low-pressure strategy using carbon-coated Al current collectors for dry-processed Li_2_S cathodes [152]. The textured carbon primer enhanced both adhesion and mechanical interlocking between the cathode layer and the Al collector, lowering the cathode/collector resistance to roughly one-fifth to one-tenth of that observed on bare Al, and enabled stable cycling at 10 MPa with ∼800 mAh g^−1^ and 78% retention over 350 cycles (Figure 6A) [152]. These studies indicate that pressure-assisted contact can be complemented by current-collector surface engineering to maintain physical interface integrity under reduced mechanical constraint.

Pressure-based strategies, however, address contact at the macroscopic cell level and do not resolve local compliance mismatches at individual particle contacts. Introducing a soft interfacial layer provides a more targeted route to bridging discontinuous solid–solid contacts and accommodating local volume-change-induced stress.

Shin and Gewirth used a localized high-concentration solvate electrolyte, composed of (MeCN)_2_–LiTFSI and a hydrofluoroether diluent, as an interfacial liquid layer in solid-state Li_2_S cells (Figure 6B) [153]. The solvate interlayer improved wetting of the Li_2_S composite cathode and established more favorable ionic contact between Li_2_S and Li_7_P_3_S_11_, leading to a discharge capacity of 760 mAh g^−1^ after 100 cycles compared with 330 mAh g^−1^ for the bare solid-state cell. Electrochemical impedance spectroscopy (EIS) and cross-sectional imaging showed that solvate permeation into cathode pores, void spaces, and solid electrolyte grain boundaries reduced interfacial resistance during cycling. This work demonstrates that soft interfacial layers can preserve contact continuity and facilitate Li^+^ transport in sulfur-based solid-state cathodes. Nevertheless, because the strategy relies on a liquid-derived interlayer, further optimization is required to balance interfacial wetting, chemical stability, and solid-state cell integrity.

Beyond localized interlayer modification, three-dimensional cathode architectures provide a structural route to maintaining contact continuity throughout the composite electrode. Jiang et al. constructed a mixed-conducting Li_2_S cathode in three steps: a Li_2_S–LiI solution was first impregnated into a carbon replica with ∼10 nm mesopores; the resulting active phase was then blended with submicron Li_6_PS_5_Br prepared by a liquid-phase route, together with vapor-grown carbon fibers as the electronic backbone [154]. In this architecture, the carbon replica provided continuous electronic pathways and space for accommodating Li_2_S/S volume changes, while the small-particle sulfide SE improved solid–solid contact and Li^+^ transport. The resulting cathode showed balanced mixed conductivity, better structural integrity than the carbon-replica-free counterpart after cycling, and reversible capacities of 1009 mAh g^−1^ after 20 cycles at 0.05 C and 650 mAh g^−1^ after 100 cycles at 0.1 C at 25 °C. These results highlight the value of 3D mixed-conducting cathode design for mitigating contact loss and maintaining coupled Li^+^/electron transport in ASSLSBs [154].

Taken together, pressure control, compliant interlayers, and three-dimensional cathode architectures demonstrate that physical interface engineering can effectively preserve solid–solid contact and maintain coupled Li^+^/electron transport in ASSLSBs. Nevertheless, improved contact alone cannot fully prevent interfacial side reactions, electrolyte decomposition, or resistive interphase formation. Therefore, physical contact stabilization must be complemented by chemical strategies that regulate interfacial reactivity and interphase chemistry.

### 4.2. Chemical Interface Engineering

Chemical interface engineering focuses on redesigning the chemical environment at electrode–solid electrolyte interfaces toward thermodynamically or kinetically stable states. Unlike physical interface engineering, which primarily addresses contact continuity, chemical strategies aim to control the composition, ion-transport properties, and mechanical stability of the interphase formed during cell assembly or cycling. In ASSLSBs, this is particularly important at both the cathode–SE interface, where sulfur species, conductive carbon, and SEs coexist, and the Li metal–SE interface, where continuous SE reduction and unstable Li deposition can occur. This section discusses chemical interface engineering in three categories: cathode–SE interface engineering, Li metal–SE interface engineering, and in situ interphase formation strategies.

#### 4.2.1. Cathode–SE Interface Engineering

Cathode–SE interface engineering focuses on regulating the chemical reactions that occur at the contact regions among sulfur species, conductive carbon, and SEs. Although cathode architecture determines how sulfur, carbon, and SE particles are spatially connected, the electrochemical stability of these contacts depends on the composition and transport properties of the interphase formed during fabrication or cycling. In sulfide-based ASSLSB cathodes, poor chemical affinity between S_8_/Li_2_S and SE particles can limit Li^+^-conductive contact, while electronically conductive carbon can accelerate oxidative decomposition of adjacent sulfide SEs. The resulting interphases may increase polarization, block Li^+^ transport, or consume active sulfur species. Therefore, cathode-side chemical interface engineering aims to construct interphases that are chemically compatible, Li^+^ conductive, and resistant to parasitic decomposition.

An early example of cathode-side interface regulation was reported by Zhu et al., who inserted a bifunctional Li^+^/electron conducting interlayer between a sulfur cathode and PEO-based SPE [158]. The interlayer, composed of SPE, conductive carbon, and a metal–organic framework (MIL-53(Al)), created a graded transition of Li^+^ and electron transport across the cathode/electrolyte boundary, thereby improving interfacial compatibility and sulfur utilization. Even with pure sulfur powder as the active material, the cell with the interlayer retained 792.8 mAh g^−1^ after 50 cycles at 0.5 C and 80 °C, compared with 291.9 mAh g^−1^ for the cell without the interlayer [158].

Building on this concept, later studies moved from physically inserted transport interlayers toward chemically generated cathode–SE interphases. Kim et al. demonstrated this approach by inducing an interfacial chemical reaction between S_8_ and LPSCl during cathode fabrication [155]. Using a weakly polar solvent, isopropyl acetate, during wet mixing promoted the formation of a polysulfido-intermediate compound, described as 3Li^+^–PS_4+n_^3−^, at the S_8_/LPSCl interface after solvent removal (Figure 6C) [155]. This interphase chemically coupled sulfur and LPSCl, improved Li^+^-conductive contact across the solid–solid interface, and enhanced sulfur utilization. The wet-mixed cathode delivered an areal capacity of 5.1 mAh cm^−2^ at 1 mA cm^−2^ and maintained stable cycling for 250 cycles at room temperature [155]. This study shows that deliberately formed cathode-side interphases can convert a poorly contacted sulfur–SE boundary into a chemically integrated reaction interface [155].

Wang et al. extended this concept by mechanochemically reacting PI_3_ with S_8_ and the chloride-rich argyrodite LPSC (Li_5.5_PS_4.5_Cl_1.5_), producing an amorphous lithium iodothiophosphate (LPSI) phase that chemically bridges the sulfur/LPSC contact during cathode fabrication [159]. Rather than acting simply as an additional cathode component, the LPSI phase served as a chemically generated interfacial bridge that reduced Li^+^ diffusion resistance and reinforced the sulfur–SE contact. The iodine-containing interphase also provided reversible redox mediation, facilitating solid-state sulfur conversion at the chemically coupled interface. The S@LPSI/LPSC cathode retained 93.8% of its capacity over 1600 cycles at a sulfur loading of 6 mg cm^−2^ and a current density of 5 mA cm^−2^ [159]. This work illustrates how cathode-side interphases can be designed as both Li^+^-conductive contact layers and reaction-mediating chemical environments [159].

Beyond these representative examples, additional cathode-side interphase strategies—including sulfur surface modifications, artificial Li^+^-conductive coatings, chemically generated sulfide/halide interlayers [160,161,162,163,164,165]—have also been explored to improve sulfur utilization while suppressing cathode-side SE decomposition. Collectively, these studies show that cathode–SE interface engineering should not be regarded simply as surface passivation, but as a strategy to chemically couple sulfur, SEs, and conductive networks into a stable and kinetically accessible reaction environment.

#### 4.2.2. Lithium Metal–SE Interface Engineering

Lithium metal–solid electrolyte interface stabilization has been extensively investigated across various all-solid-state battery chemistries and has been comprehensively discussed in several dedicated reviews [35,166,167,168]. Therefore, this section focuses on representative strategies directly demonstrated in ASSLSB configurations. In this context, chemical interface engineering aims to construct artificial or modified interphases that suppress parasitic reactions at the Li/electrolyte interface while maintaining Li^+^ transport, mechanical integrity, and compatibility with sulfur-derived species.

In polymer and quasi-solid-state Li–S systems, anode-side interface modification has been used to improve Li^+^ transport while suppressing polysulfide-induced side reactions at the Li surface. Liu et al. grafted Tween-20 (a polymeric molecule containing ethylene oxide segments and alkyl chains) onto Li metal to form a polymer-rich interfacial layer, in which oxyethylene groups facilitated Li^+^ transport and alkyl chains limited polysulfide access to metallic Li [169]. The resulting Tween-grafted Li enabled improved PEO-based Li–S cell performance, delivering 1051.2 mAh g^−1^ at 0.2 C and stable cycling for 500 cycles at 2 C [169]. A related SPE-side strategy was reported by Fan et al., who deposited an ultrathin amorphous Al_2_O_3_ layer on PEO–LiTFSI SPEs by atomic layer deposition [170]. The Al_2_O_3_ coating increased the Li^+^ transference number, improved Li metal compatibility, and suppressed dendrite formation for more than 500 h in Li symmetric cells. Because the coated SPE also reduced polysulfide migration and self-discharge, these studies show that Li-side interface engineering in polymer-based Li–S cells must address both Li plating stability and sulfur-species crossover [170].

For sulfide SE-based ASSLSBs, artificial inorganic interphases have been directly employed to suppress reductive SE decomposition at the Li metal interface. Duan et al. demonstrated this concept by constructing an artificial LiI interphase at the Li/LGPS interface using chemical iodine–vapor deposition (Figure 6D) [156]. In this process, iodine vapor reacted with lithium metal to form a dense LiI layer composed of interwoven nano-LiI crystals. The LiI interphase combined chemical stability, Li^+^ conductivity, electronic insulation, and mechanical toughness, thereby blocking direct Li/LGPS contact while maintaining interfacial ion transport. When applied to ASSLSBs, the LiI-modified cell delivered 1360 mAh g^−1^ at 0.2 mA cm^−2^ and retained 80.6% of its capacity after 150 cycles [156]. This work highlights that Li metal–SE interface engineering in ASSLSBs should address both chemical compatibility and mechanical durability during repeated Li plating/stripping [156].

Beyond halide-based interphases, Wu et al. proposed Li_2_S as a single-ion-conducting artificial SEI for stabilizing the Li/LGPS interface [171]. A thin Li_2_S layer was formed on Li metal by chemical vapor deposition using sulfur vapor, producing an electronically insulating but Li^+^-conductive interphase. The Li_2_S interphase provided a chemically compatible buffer between Li metal and LGPS, suppressing electron-driven LGPS reduction while enabling more stable Li plating/stripping. Li/Li_2_S/LGPS symmetric cells operated for more than 500 h at 0.15 mA cm^−2^, and the corresponding ASSLSB showed 90.8% capacity retention after 100 cycles at 0.2 mA cm^−2^ [171]. This example demonstrates that sulfide-derived single-ion-conducting interphases can stabilize Li metal contacts while preserving chemical continuity with sulfide-based ASSLSB architectures.

These examples highlight that Li metal–SE interface engineering in ASSLSBs should not be limited to dendrite suppression alone. Effective anode-side interphases must simultaneously prevent electron leakage, maintain Li^+^ transport, suppress SE decomposition, and limit parasitic reactions with sulfur-derived species. Future designs should therefore prioritize chemically compatible, mechanically robust, and minimally resistive artificial interphases that remain stable under repeated Li plating/stripping and high-energy sulfur cathode operation.

#### 4.2.3. In Situ Interphase Formation and Stabilization

Whereas the preceding sections focus on interphases introduced during material synthesis or cell fabrication, in situ interphase formation forms adaptive layers during the initial electrochemical cycling. This strategy is attractive for buried solid–solid interfaces, where uniform ex situ coating is difficult and where the interphase must evolve under operating potentials, volume changes, and local current distributions. By forming the protective or ion-conductive phase under working conditions, in situ strategies can improve interfacial conformity and chemical compatibility without requiring complete pre-coating of each particle.

An early example of formation-assisted interface stabilization was reported by Umeshbabu et al., who introduced a small amount of LiTFSI/PYR_13_TFSI ionic liquid at the Li/LGPS interface [172]. The ionic liquid improved interfacial wetting and promoted the formation of an in situ SEI layer on Li, lowering the initial interfacial resistance of Li/LGPS/Li symmetric cells from 2021 Ω cm^2^ for the unmodified interface to 142 Ω cm^2^ for the IL-modified interface. During open-circuit storage, the interfacial resistance initially increased as the passivation layer formed and then stabilized, indicating the development of a more stable Li/LGPS interface. The modified cells further showed stable Li stripping/plating for 1200 h at 0.038 mA cm^−2^ and 1000 h at 0.1 mA cm^−2^, demonstrating that liquid-assisted in situ passivation can stabilize reactive sulfide SE/Li interfaces [172].

Formation-based interfacial stabilization has also been extended to cathode-side interfaces. Li et al. employed a pre-cycling thermal formation process to stabilize the LiBH_4_/S interface before electrochemical operation [173]. Heating at 155 °C induced a partial reaction between LiBH_4_ and sulfur, producing a Li_2_B_12_H_12_/Li_2_S-containing passivating interphase that reduced cathode–electrolyte reactivity and broadened the effective stability window of the hydride electrolyte. Compared with the untreated cell, the thermally formed ASSLSB showed slower capacity decay and retained 587 mAh g^−1^ after 100 cycles, indicating that pre-stabilizing the cathode–SE interphase can improve cycling stability in hydride-based ASSLSBs [173].

A more recent example of electrochemically driven interphase formation was reported by Li et al., who introduced a KBr-containing eutectogel molecular layer (KEGML) to trigger electrochemically driven interphase formation at the cathode–SE interface (Figure 6E) [157]. The controlled reaction between a P_2_S_5_ pre-interphase and Li^+^ generated a gradient Li_3_PS_4_-rich interphase at the sulfur cathode interface, suppressing direct carbon–LPSCl contact, mitigating LPSCl decomposition, and promoting more uniform Li^+^ transport. KEGML increased the ionic transport capability by approximately eightfold after 200 cycles and enabled 1578.9 mAh g^−1^ with 99.9% capacity retention over approximately 1.5 years, as well as a high areal capacity of 13 mAh cm^−2^ over 200 cycles [157].

Formation-based interface engineering is therefore most valuable for buried ASSLSB interfaces that are difficult to access by conventional ex situ coating. Rather than relying on pre-formed layers alone, these strategies generate stabilizing interphases through liquid-assisted passivation, thermal treatment, or electrochemical conditioning. However, their practical effectiveness requires precise control of formation conditions, because excessive interphase growth can consume Li inventory, active sulfur, or electrolyte components and increase interfacial resistance. Future work should therefore prioritize self-limiting formation chemistries that create thin, Li^+^-conductive, and chemically stable interphases under realistic cycling conditions. Representative ASSLSB cell configurations spanning the cathode, solid-electrolyte, and interface strategies discussed in this review, together with their key cell-level performance metrics where reported, are summarized in Table 1.

**Table 1:** Representative ASSLSB cell configurations reported in the literature, organized by the cathode, solid-electrolyte, and interface strategies discussed in this review. Values are taken directly from the original reports; entries not specified are marked N/A.

Section	Cathode Composite	Active Material Loading (mg cm^−2^)	Solid Electrolyte Type	Capacity * at 1^st^ Discharge (mAh g^−1^)	Capacity * at n^th^ Cycle (mAh g^−1^)	Cell Type	Ref.
Sulfur /Carbon Composite Cathodes (2.1)	S/carbon black/Li_7_P_2.9_Mn_0.1_S_10.7_I_0.3_	N/A	Li_7_P_2.9_Mn_0.1_S_10.7_I_0.3_	796 (0.05 C)	~800 (60th, 0.05 C)	ASS	[52]
	S/CMK-3/Li_3.25_Ge_0.25_P_0.75_S_4_	N/A	Li_3.25_Ge_0.25_P_0.75_S_4_	3239 (0.09 C)	1300 (20th, 0.09 C)	ASS	[62]
	S/C (interconnected mesoporous carbon)/Li_3_PS_4_	2.6 (S)	Li_3_PS_4_	1200 (1.3 mA cm^−2^)	1100 (400th, 1.3 mA cm^−2^)	ASS	[63]
	S/KB/Li_3_PS_4_	1.5 (S)	Li_3_PS_4_	1793 (0.1 C)	1437 (100th, 0.1 C)	ASS	[59]
	S@CNTs/AB/LGPS	0.45 (S)	LGPS + 75%Li_2_S-24%P_2_S_5_-1%P_2_O_5_	1431 (0.1 C)	660.3 (400th, 1 C)	ASS	[64]
	S@rGO/AB/LGPS	0.45 (S)	LGPS + 75%Li_2_S-24%P_2_S_5_-1%P_2_O_5_	1526 (0.05 C)	830 (750th, 1 C)	ASS	[59]
	S/PPCF/LPSCl	N/A	LPSCl	1166 (0.05 C)	710 (220th, 0.1 C)	ASS	[70]
	S/PPy@NCNT/AB/Li_7_P_3_S_11_	4.5 (S)	Li_7_P_3_S_11_	1348 (0.1 C)	715 (250th, 0.2 C)	ASS	[60]
	S–CNG/Li_5.5_PS_4.5_Cl_1.5_	1.5–7.6 (S)	Li_5.5_PS_4.5_Cl_1.5_	2.6 mAh cm^−2^ (0.1 C)	~2.1 mAh cm^−2^ (235th, 0.1 C)	ASS	[72]
Alternative Composite Cathodes (2.2)	Li_2_S–LPSCl–C/80Li_2_S·20P_2_S_5_	3.6 (Li_2_S)	80Li_2_S·20P_2_S_5_ glass-ceramic	648 (50 mA g^−1^)	830 (60th, 50 mA g^−1^)	ASS	[74]
	Li_2_S–LiVS_2_/Li_5.5_PS_4.5_Cl_1.5_	4.0 (Li_2_S + LiVS_2_)	Li_5.5_PS_4.5_Cl_1.5_	1.8 mAh cm^−2^ (1 mA cm^−2^)	~1.8 mAh cm^−2^ (500th, 1 mA cm^−2^)	ASS	[73]
	Li_2_S–Li_x_In_2_S_3_–VGCF/Li_7_P_3_S_11_	4.0 (Li_2_S)	Li_7_P_3_S_11_	3.47 mAh cm^−2^ (0.192 mA cm^−2^)	2.86 mAh cm^−2^ (200th, 0.192 mA cm^−2^)	ASS	[77]
	S–TiS_2_–BP2000–CNT/LPSCl	4.0 (S + TiS_2_)	LPSCl	7.05 mAh cm^−2^ (0.1 C)	6.84 mAh cm^−2^ (150th, 0.1 C)	ASS	[81]
	S–VS_2_/Li_3_PS_4_	15.5 (S + VS_2_)	Li_3_PS_4_	7.8 mAh cm^−2^ (0.12 mA cm^−2^)	5.2 mAh cm^−2^ (10th, 0.12 mA cm^−2^)	ASS	[82]
	SPAN/carbon black/PVDF	~1 (SPAN)	LiTFSI:PEO /LATP /LiTFSI:PEO	1793 (0.1 C)	784 (120th, 0.1 C)	QSS	[96]
	p(S-DVB)/KB/LiFSI:PEO	0.9–1.1 (S)	LiFSI:PEO	1100 (0.05 C)	650 (50th, 0.1 C)	QSS	[99]
	Li_2_S/AQT/LiTFSI:PEO	0.2–0.7 (Li_2_S)	LiTFSI:PEO	1133 (0.1 C)	997 (20th, 0.1 C)	QSS	[83]
Inorganic Solid Electrolytes (3.1)	Li_2_S/VGCF/Li_7_Sb_0.05_P_2.95_S_10.5_I_0.5_	3.5 (Li_2_S)	Li_7_Sb_0.05_P_2.95_S_10.5_I_0.5_	622 (0.06 mA cm^−2^)	687 (15th, 0.06 mA cm^−2^)	ASS	[109]
	S@KB/Super P/LBPSI	1.1 (S)	LBPSI	823 (5 C)	80.2% (25,000th, 5 C)	ASS	[110]
	S@CNT/Li_6.5_La_3_Zr_1.5_Nb_0.5_O_12_	5.4 (S)	Li_6.5_La_3_Zr_1.5_Nb_0.5_O_12_	~1200 (50 mA g^−1^)	— (50th, low capacity loss)	QSS	[115]
	S/C/catholyte	N/A	LLZTO	N/A	805 (500th, 1 C)	QSS	[116]
	S@CNT	N/A	Li_3_HoBr_6_	582 (0.1 C)	— (400th, 0.2 C, stable cycling)	ASS	[120]
	S@(KB+Maxsorb C)/PVDF	0.12 (S)	LiBH_4_@MCM-41	~3100 (1st, 0.03 C)	1220 (40th, 0.03 C)	ASS	[122]
Polymer Electrolytes (3.2)	SPAN/CNT/PVDF-HFP·LiFSI	0.5–1.0 (S)	PVDF-HFP/LiFSI (1:1) + 13 wt% DMF	N/A	897.4 (100th, 0.2 C)	QSS	[128]
	S-C/Super P/PVDF	~1 (S)	PEO/PIM-1 (8 wt%)/LiTFSI	1181 (0.5 C)	730 (100th, 0.5 C)	QSS	[129]
	SPAN/carbon black/PTMG-HDI-BHDS·LiFSI/PVDF	2.0–2.2 (SPAN)	PTMG-HDI-BHDS/LiFSI	602 (0.3 C)	560 (700th, 0.3 C)	QSS	[130]
	S@C/carbon black/EMS	0.5 (S)	TEGDME gel composite	≈1100 (0.1 C)	680 (200th, 0.3 C)	QSS	[134]
	S-C	≈5 (S)	COF-SH@PVDF-HFP gel	808.4 (2 C)	568.8 (800th, 2 C)	QSS	[135]
	S@BP-2000/AB/PVDF	N/A	PEO-PAN-LiTFSI	1200 (0.1 C)	— (75th, 0.1 C, stable cycling)	QSS	[136]
Hybrid Composite Electrolytes (3.3)	S@LLZO@C/PEO-LiClO_4_	1.2 (S)	LLZO-PEO-LiClO_4_	>900 (0.05 mA cm^−2^)	98.7% (90th, 0.05 mA cm^−2^)	QSS	[143]
	SPAN/carbon black/PEO-LiTFSI	N/A	LGPS/PEO composite	1772 (0.1 C)	588 (50th, 0.1 C)	QSS	[144]
	S@C/CNT/LA133	1.5 (S)	Vr/PEO laminar composite	1254 (0.05 C)	1017 (150th, 0.05 C)	QSS	[145]
	S/KB/BN-PEO-PVDF	≈0.9 (S)	BN-PEO-PVDF	≈1200 (0.05 C)	≈790 (50th, 0.1 C)	QSS	[149]
	S@KB	N/A	MIL-125–NH_2_ + PAN + PEO + LiTFSI	≈418.1 (0.5 C)	>323.2 (400th, 0.5 C)	QSS	[150]
	SPAN	N/A	PVDF + polyurethane + LLZTO	2194.8 (0.1 C)	998.3 (350th, 0.5 C)	QSS	[146]
Physical Interface Engineering (4.1)	Li_2_S/AB/PTFE	N/A	LPSCl	799 (0.33 C)	78% (350th, 0.33 C)	ASS	[152]
	Li_2_S/KB/Li_7_P_3_S_11_	0.57–0.75 (Li_2_S)	Li_7_P_3_S_11_	990 (0.1 C)	760 (100th, 0.1 C)	QSS	[153]
	Li_2_S–LiI/CR10/Li_6_PS_5_Br/VGCF	>2 (Li_2_S)	Li_3.45_Ge_0.45_P_0.55_S_4_	785 (0.1 C)	650 (100th, 0.1 C)	ASS	[154]
Chemical Interface Engineering (4.2)	S/Super P/PAA	0.7 (S)	PEO–LiTFSI (+ MIL-53(Al))	1457 (0.5 C)	792.8 (50th, 0.5 C)	QSS	[158]
	S/MoS_2_@CNT/LPSCl	4 (S)	LPSCl	5.1 mAh cm^−2^ (1 mA cm^−2^)	5.1 mAh cm^−2^ (250th, 1 mA cm^−2^)	ASS	[155]
	S/LPSI interlayer/Li_5.5_PS_4.5_Cl_1.5_	6 (S)	Li_5.5_PS_4.5_Cl_1.5_	5.79 mAh cm^−2^ (0.5 C)	5.4 mAh cm^−2^ (1600th, 0.5 C)	ASS	[159]
	S/Super P	N/A	PEO–LiTFSI	1051.2 (0.2 C)	801.3 (150th, 1 C)	QSS	[169]
	S/PAN-coated carbon	N/A	PEO–LiTFSI + Al_2_O_3_ ~5 nm	1306 (0.2 C)	680 (200th, 0.2 C)	QSS	[170]
	S/KB/LGPS	0.45 (S)	LGPS	1360 (0.2 mA cm^−2^)	80.6% (150th, 0.2 mA cm^−2^)	ASS	[156]
	S/MWCNTs/LGPS	0.45 (S)	LGPS	921.1 (0.2 mA cm^−2^)	836.8 (100th, 0.2 mA cm^−2^)	ASS	[171]
	S@KB/AB/LGPS	1.28 (S)	LGPS	1017 (83.5 mA g^−1^)	726 (50th, 83.5 mA g^−1^)	QSS	[172]
	S(thermal-formed)/C/LiBH_4_	N/A	LiBH_4_	1455 (0.03 C)	587 (100th, 0.1 C)	ASS	[173]
	S/KB@P/LPSCl	1 (S)	LPSCl	1579 (0.1 C)	1579 (≈550th, 0.15 C)	ASS	[157]

* Capacities are given in mAh g^−1^ unless an areal capacity (mAh cm^−2^) is noted, with the rate or current density in parentheses. Cells are classified as all-solid-state (ASS) when both the separator and the cathode ionic phase are solid, and as quasi-solid-state/hybrid (QSS) when a small quantity of liquid electrolyte, catholyte, gel phase, or liquid-assisted interfacial treatment is present.

## 5. Conclusions and Future Perspectives

This review has examined recent progress in cathodes, solid electrolytes, and electrode–electrolyte interfaces of ASSLSBs. At the cathode level, structured carbon hosts—mesoporous frameworks, one-dimensional carbon networks, and two-dimensional architectures—and surface-engineered conductive networks improve triple-phase boundary density, volume-change tolerance, and SE compatibility beyond what simple ball-milled composites achieve. Alternative active materials including Li_2_S, metal sulfides, organosulfur compounds, and molecular redox mediators offer distinct routes to improved conversion kinetics and chemo-mechanical stability. At the electrolyte level, sulfide SEs remain dominant for their high conductivity and cold-processability, while halide and hybrid composite electrolytes have emerged as competitive alternatives that better balance electrochemical stability, interfacial contact, and processability. At the interface level, both physical and chemical engineering strategies confirm that solid–solid interfacial resistance—not bulk SE conductivity—is the primary performance-limiting factor in ASSLSBs.

Several challenges remain unresolved, and they are best viewed as coupled contributions to the overall cell resistance rather than as independent problems. Because the bulk SE conductivity is rarely the limitation, the effective ionic conductivity within the composite cathode is decisive. This effective value is substantially lower than the bulk conductivity and worsens with increasing sulfur loading. Beyond this transport limitation, the charge-transfer resistance at the solid–solid reaction front and the chemo-mechanical contact loss during cycling add further resistance in series and reinforce one another, because contact loss exposes fresh SE surface that accelerates interfacial decomposition, while the resulting resistive interphases intensify the local stress that drives further contact loss. A related trade-off appears in composite design, where finer mixing improves triple-phase contact and sulfur utilization but enlarges the reactive SE surface area. Because the cell is governed by whichever contribution dominates, these factors must be addressed together. Processing scalability, including low-pressure fabrication and roll-to-roll compatible SE film deposition, remains a critical gap between laboratory demonstration and practical cell formats.

Future progress requires treating the cathode, electrolyte, and interface as a single coupled system rather than optimizing each independently. The cathode composite sets the local transport demands that determine the conductivity and deformability required of the electrolyte, and the chemical and mechanical compatibility between them governs whether a stable interface can form. SE materials with simultaneously high conductivity, chemical compatibility with carbon additives, and stability against the lithium metal anode would address the fundamental bottleneck in cathode composite design. Interface engineering strategies should evolve toward self-limiting in situ chemistries that generate protective interphases under realistic cycling conditions without consuming Li inventory or active sulfur.

Progress should also be benchmarked against cell-level metrics rather than material-level capacity alone. Practically relevant ASSLSBs will need a sulfur content near 50 wt% and an areal loading of roughly 4 to 6 mg cm^−2^, combined with a high sulfur utilization corresponding to a discharge capacity of at least ~1200 mAh g^−1^, so that the areal capacity reaches the ~6 mAh cm^−2^ range. They will also require thin solid-electrolyte separators (below ~50 μm, and ideally near 30 μm), a lean lithium-metal anode with a negative-to-positive capacity ratio close to unity, low operating stack pressure (on the order of 10 MPa or below), and near-room-temperature operation. Meeting these targets together would raise the projected gravimetric and volumetric energy densities toward ~500 Wh kg^−1^ and ~500 Wh L^−1^, surpassing current lithium-ion cells.

These directions can be prioritized along a near-term to mid-term timeline rather than tackled all at once. In the near term, the priority is to reduce the dominant interfacial and transport losses by stabilizing the cathode–SE and lithium metal–SE interfaces through self-limiting in situ interphases, by raising the effective ionic conductivity of the composite cathode while preserving contact at higher sulfur loading, and by selecting high-conductivity sulfide or halide SEs that are chemically compatible with carbon and lithium metal. In the mid term, the focus shifts to translation, namely scalable and low-pressure fabrication of thin SE films and their integration into pouch-format cells that meet the cell-level targets together. The operando characterization discussed below underpins both stages, providing the mechanistic feedback needed to guide material and interface choices.

Although mechanistic investigation is beyond the scope of this review, advanced characterization tools—including operando X-ray diffraction and tomography, cryogenic electron microscopy—are essential for resolving degradation processes at buried solid–solid interfaces, elucidating sulfur speciation during cycling, and providing the quantitative mechanistic feedback needed to guide rational material and interface design. Continued progress in these coupled materials and interface design directions will determine whether ASSLSBs can move from high-promise laboratory systems toward practically relevant high-energy cells.

## Data Availability

No new data were created or analyzed in this study. Data sharing is not applicable to this article.
